# Supplementary material for: STOPS approach to individualised physiotherapy versus usual physiotherapy care for chronic low back pain in India: A randomised controlled trial protocol
Source: PLoS One. 2025 Dec 30;20(12):e0339280. doi: 10.1371/journal.pone.0339280 (PMC12752971; doi:10.1371/journal.pone.0339280)
Supplement: S1 File — (DOCX) [file pone.0339280.s001.docx]

**Research proposal for Ethical clearance**

| **Title** | Individualized physiotherapy versus usual care (STOPS trial) for Low Back Disorder in Indian primary care: A Randomized Clinical Trial |
| --- | --- |
| **Name and address of the principal investigator** | Dr. Kavitha Raja, PT, PhD  Professor and principal  JSS College of Physiotherapy, MG Road, Mysuru-4 |
| **Name of the institution** | JSS college of Physiotherapy, Mysuru |
| **Project details** | Submission for Ethical Approval |
| **Date** | 30/01/2024 |

| **Sl.No** | **Title** | **Page no** |
| --- | --- | --- |
| 1. | Introduction | **3-5** |
| 2. | Literature review | **6-11** |
| 3. | **Methodology**   - Study design - Sampling and sample size - Materials - Outcome measures - Procedure | **12-24** |
| 4. | **Data analysis** | **24-26** |
| 5. | **Bibliography** | **26-41** |
| 6. | **Annexure**  Physical examination  Patient reported outcome measures  Subgroup classification and treatment  Qualitative Interview  FMRI  Informed consent | **41-91**  41-45  47-66  67-77  78-86  87  88-89 |

## **Introduction**

Low back pain (LBP) is a significant issue with considerable impact on people’s lives and economies. From 1990 to 2019, the global incidence of LBP increased by 50% ^[1]^, and is now the leading cause of disability worldwide. It has remained top-ten-ranking causes of disability adjusted life years (DALY) ^[2]^. In 2019, it was estimated 223.5 million people have LBP and 63.7 million LBP-related DALYs worldwide.^[1,2]^ South Asia is also home to the highest number of people with LBP (96.3 million) among all regions of the world ^[3]^ and India being the largest country in South Asia accounts nearly about 8% people yearly lived with disability (YLD) due to LBP.^[4]^ LBP is the leading health condition contributing to the need for rehabilitation services ^[5]^ and it is estimated that the number of people with low back pain will increase in the future, and even more rapidly in low-income and middle-income countries.^[6]^

The underlying mechanisms of symptom onset and progression are highly variable, complicated and heterogeneous in low back pain, thus treatment approaches to lessen LBP have proven to be extremely difficult.^[7,8]^ .Various methods (or approaches) have been proposed and tried to optimize clinical decision making in LBP, such as the use of classification systems or CPRs with varying degrees of success^[7,9-17]^. Despite this, there is a global evidence-to-practice gap for treating LBP, and these treatment guidelines are not being adopted as recommended. ^[6,18-23]^

Consistent recommendations from international guidelines have been made in recent years, indicating conservative physiotherapy intervention is useful in treating LBP. Individuals with acute LBP should get conservative treatments including encouragement to stay active, education about the benign nature of LBP, and assurances regarding the absence of significant pathology.^[10,24-35]^ However, treatment based on the biopsychosocial model of care should be given to people who are at risk of acquiring chronic pain and impairment. ^[7,8,10,22-29]^ and also patients with CLBP.^[36]^ Also, in CLBP its is more flexible, multidimensional, clinically-reasoned approach to profile patient complexity may be required to inform individualised, patient-centred care.^[37]^ which can lead to healthcare cost savings and better return to work and by implication a better economical outcome for the patient^.[38]^

Although, various randomized controlled trials (RCT) are have been conducted on variety of classification and stratification system on LBP like Start back approach, Treatment-Based Classification (TBC) , Mechanical Diagnosis and Therapy (MDT), the Cognitive Functional Therapy (CFT), Movement System Impairment (MSI) or, Pathoanatomical-Based Classification (PBC),^[12,39-43]^ , there is currently insufficient evidence supporting the use of any classification systems for managing LBP in clinical practice.^[10, 44]^ The randomized controlled trials (RCTs) upon which guideline recommendations are based typically show small effect sizes of questionable clinical importance ^[18,32,44,45].^ A potential reason for the limited effects demonstrated in RCTs is at misunderstandings that NSLBP is a homogeneous group. ^[46,47]^

While the STarT back approach and Cognitive Functional Therapy (CFT) integrate risk stratification as a viable strategy to screen for the risk of patients experiencing poor outcomes in terms of pain and disability based on psychosocial variables, the Mechanical Diagnosis and Therapy (MDT), Pathoanatomical-Based Classification (PBC), and Movement System Impairment (MSI) approaches primarily treat mechanical dysfunction regardless of psychosocial presentation. Recent guidelines, however, encouraged, to incorporate the treatment- and mechanism-based categorization systems as well as psychological component throughout the patient's examination. ^[10,48]^ Thus, most the treatment strategies fail to consider the multidimensional nature of LBP^[7,8,10,22-29,36-39]^ and patho-anatomical factors despite emerging evidence, that they may be important in clinical decision-making.^[49-53]^

Therefore, recent guidelines have highlighted drawbacks that necessitate the consideration of a classification system that combines the biomechanical and psychological aspects of LBP in a more comprehensive way. The value of individualized or personalized interventions includes education, counselling, exercise, mobility training, and manual therapy, depending on the patient's clinical features and examination ^[10,48,54]^ and recommend decision making to have interim evaluations that should be scheduled to track patient progress and advise the revision of the intervention strategies and tactics accordingly. ^[10]^ Explicit information regarding the dosage, duration, and other aspects of how the indicated treatments should be administered should also be provided. ^[55]^

The Specific Treatment Of Problems of the Spine (STOPS) is a classification system of LBP that is based on the biopsychosocial model of care.^[56]^ The basic premise of the STOPS approach is that LBP is multifactorial, and factors other than the pathoanatomical source of pain (e.g., nociceptive source of symptoms) need to be considered in a robust classification system including neurophysiological (e.g., central sensitization) and psychosocial components (e.g., fear-avoidance) of the pain.^[56]^ Based on this philosophy, the STOPS approach was developed where patients are sub-grouped and treatment strategies are individualised to the participant’s presentation ^[56-60]^ and series of studies has been investigated in Australia and has been proven to be effective in a subgroup of individuals with LBP in that country. ^[14,61-65]^

**Need for the study:**

The STOPS approach is yet to be investigated in a different country and setting than where it was initially developed. Research has showed evidence-to-practice gap for treating LBP, and these treatment guidelines are not being adopted as recommended in India. ^[18,20]^ Despite the high incidence of LBP, where caste, economic status, religion, and even geographic location (rural or urban) are recognized socio-cultural barriers to healthcare seeking, India lacks effective and targeted treatment methods based on the biopsychosocial model of care, which exacerbates disability and widens the gap between India and affluent countries in terms of LBP care.^[4,5,6,18]^  These findings indicate that the quality of physiotherapy care for LBP in India needs to be optimized.

## LITERATURE REVIEW

| Sl. No | 1 |
| --- | --- |
| Author | Muhammed Rashid, T. Muhammad, Shobhit Srivastava |
| Tittle | Back pain and treatment seeking among community-dwelling older adults: Findings from a population-based survey. |
| Objective | To explore the prevalence and factors associated with back pain and types of treatment seeking of older people with back pain. |
| Study | Cross-sectional design has been conducted using a population-based survey of the Longitudinal Ageing Study in India |
| Sample size | 31,464 older adults |
| Outcome measure | Questionnaire used to assess back pain and treatment seeking |
| Results and  Conclusion | Out of the participants experiencing back pain, approximately 34% sought relief. Among them, the majority, accounting for 46.2%, opted for external applications, followed by 40.8% who turned to analgesics, and only 6.6% who pursued therapy. Notably, older adults with a higher level of education were more likely to choose therapy compared to those with little or no education. Furthermore, participants from the wealthiest quintile demonstrated a greater inclination towards therapy compared to those from the lowest quintile. Despite being globally recommended as the primary approach to managing back pain, therapy was the least utilized method and should be actively promoted. |

| Sl. No | 2 |
| --- | --- |
| Author | Supreet Bindra, Sinha A.G.K, and Benjamin A.I |
| Tittle | Epidemiology of Low Back Pain in Indian Population: A Review |
| Objective | Describing the epidemiology of LBP in terms of prevalence, demographic features, risk factors, impact and health care service utilization for LBP in Indian context |
| Study | Review |
| Search engine | Databases such as Pub med, Research gate and Google scholar |
| Conclusion | When it comes to seeking healthcare services for lower back pain (LBP), a significant number of individuals chose not to consult any professional. The next commonly adopted approach was self-medication with over-the-counter drugs, while a majority of people preferred traditional treatments over the allopathic system of medicine. Factors such as low socioeconomic status, limited education, past history of LBP, physical aspects like lifting heavy objects, engaging in repetitive or static postures, and psychosocial factors such as anxiety, depression, job dissatisfaction, lack of control over work, mental stress, long working hours, and obesity were found to be associated with LBP. However, it should be noted that the existing literature on LBP in the Indian population lacks conclusive evidence due to various reasons. These include small sample sizes in studies, lack of consistency in defining LBP, diversity among the populations under study, insufficient epidemiological research in the general population, and inadequate analysis of risk factors, which restricts the generalizability of the findings. |

| Sl. No | 3 |
| --- | --- |
| Author | Joshua Zadro, Mary O’Keeffe, Christopher Maher |
| Tittle | Do physical therapists follow evidence-based guidelines when managing musculoskeletal conditions? Systematic review |
| Objective | To determine what percentage of physical therapy treatment choices for musculoskeletal conditions agree with management recommendations in evidence-based guidelines and systematic reviews. |
| Study | Systematic review. |
| Sample size | Performed searches in Medline, Embase, Cumulative Index to Nursing and Allied Health Literature, Cochrane Central Register of Controlled Trials, Allied and Complementary Medicine, Scopus and Web of Science combining terms synonymous with ‘practice patterns’ and ‘physical therapy’ from the earliest record to April 2018. Studies that quantified physical therapy treatment choices for musculoskeletal conditions through surveys of physical therapists, audits of clinical notes and other methods. Included 94 studies |
| Outcome measure | Using medians and IQRs, they summarised the percentage of physical therapists who chose treatments that were recommended, not recommended and had no recommendation, and summarised the percentage of physical therapy treatments provided for various musculoskeletal conditions within the categories of recommended, not recommended and no recommendation. Results were stratified by condition and how treatment choices were assessed (surveys of physical therapists vs audits of clinical notes). |
| Results and  Conclusion | Many physical therapists seem not to follow evidence-based guidelines when managing musculoskeletal conditions. There is considerable scope to increase use of recommended treatments and reduce use of treatments that are not recommended. |

| Sl. No | 4 |
| --- | --- |
| Author | Nadia Corp, Gemma Mansell, Siobhán Stynes, Gwenllian Wynne-Jones, Lars Morsø, Jonathan C. Hill, Danielle A. van der Windt |
| Tittle | Evidence-based treatment recommendations for neck and low back pain across Europe: A systematic review of guidelines |
| Objective | To synthesized evidence from European neck and low back pain (NLBP) clinical practice guidelines (CPGs) to identify recommended treatment options for use across Europe |
| Study | Systematic review |
| Sample size | Comprehensive searches of thirteen databases were conducted, from 1st January 2013 to 4th May 2020 to identify up-to-date evidence-based European CPGs for primary care management of NLBP, issued by professional bodies/organizations |
| Outcome measure | The AGREE II checklist was used to critically appraise guidelines. |
| Results and  Conclusion | Low back pain the guidelines recommended entirely non-pharmacological treatments, additionally including work-based interventions, advice/programmes to return to work for specific subgroups |

| Sl. No | 5 |
| --- | --- |
| Author | Nafisa Fidvi & Stephen May2 |
| Tittle | Physiotherapy Management of Low Back Pain in India- A Survey of Self-reported Practice |
| Objective | To explore the self-reported management strategies employed by physiotherapists in India as it is unknown if these are in line with contemporary guidelines. |
| Study | Cross-sectional survey study |
| Sample size | 350 physiotherapists |
| Outcome measure | self-completed questionnaire |
| Results | All therapists reported that they gave some kind of advice to patients, used exercises and electrotherapy, and in addition about half used manual therapy. The majority of therapists used 8–12 sessions of treatment. |
| Conclusion and limitations | Indian physiotherapy management of low back pain has shown several areas of ‘good practice’ in line with contemporary guidelines. It also highlighted potential areas of concern regarding evidence-based practice; namely, very common use of passive electrotherapy modalities and potential excessive treatment. This report has implications for physiotherapy practice and education in India.  limitation of this study is that it does not characterize between cases of  Acute and chronic LBP according to guideline recommendation. These findings were from one of state i.e. Maharashtra. |

| Sl. No | 6 |
| --- | --- |
| Author | Jon J Ford, Andrew J Hahne, Luke D Surkitt, Alexander Y P Chan, Matthew C Richards, Sarah L Slater, Rana S Hinman, Tania Pizzari, Megan Davidson, Nicholas F Taylor |
| Tittle | Individualised physiotherapy as an adjunct to guideline-based advice for low back disorders in primary care: a randomised controlled trial |
| Objective | This study investigates whether individualised physiotherapy plus guideline-based advice results in superior outcomes to advice alone in participants with low-back disorders. |
| Intervention | prospective parallel group multicentre randomised controlled trial was set in 16 primary care physiotherapy practices in Melbourne, Australia. Random assignment resulted in 156 participants receiving 10 sessions of physiotherapy that was individualised based on pathoanatomical, psychosocial and neurophysiological barriers to recovery combined with guideline-based advice, and 144 participants receiving 2 sessions of physiotherapist-delivered advice alone. |
| Sample size | Sample size 300 |
| Outcome measure | Primary outcomes were activity limitation (Oswestry Disability Index) and numerical rating scales for back and leg pain at 5, 10, 26 and 52 weeks postbaseline. Analyses were by intention-to-treat using linear mixed models. |
| Results | Between-group differences showed significant effects favouring individualised physiotherapy for back and leg pain at 10 weeks (back: 1.3, 95% CI 0.8 to 1.8; leg: 1.1, 95% CI 0.5 to 1.7) and 26 weeks (back: 0.9, 95% CI 0.4 to 1.4; leg: 1.0, 95% CI 0.4 to 1.6). Oswestry favoured individualised physiotherapy at 10 weeks (4.7; 95% CI 2.0 to 7.5), 26 weeks (5.4; 95% CI 2.6 to 8.2) and 52 weeks (4.3; 95% CI 1.4 to 7.1). Responder analysis at 52 weeks showed participants receiving individualised physiotherapy were more likely to improve by a clinically important amount of 50% from baseline for Oswestry (relative risk (RR=1.3) 1.5; 95% CI 1.2 to 1.8) and back pain (RR 1.3; 95% CI 1.2 to 1.8) than participants receiving advice alone. |
| Conclusion | 10 sessions of IPA resulted in significantly better outcomes than 2 sessions of guideline-based advice for Oswestry at 10, 26 and 52 weeks as well as back and leg pain at 5, 10 and 26 weeks. The size of the effect for primary outcomes was less than the MCID. However, based on our preplanned analysis, IPA resulted in differences compared with advice including rapid reductions in pain and durable improvements in activity limitation that are also likely to be clinically important. |

## Methods

## 2.1 Study design

This study is blinded, two armed parallel Randomized Controlled Trial (RCT), will examine the effectiveness of Specific Treatments for the Problems of Spine (STOPS) Physiotherapy care for the management of Low Back Pain (LBP) in comparison with standard physiotherapy (usual care).An overview of process of study is presented in Figure 1.

Assessed for eligibility

(n = …)

# Enrollment

Excluded (n = …) Not meeting inclusion criteria

(n = …)

Refused to participate (n = …)

Other reasons (n = …)

Randomized (n = 150)

Allocated to Usual care intervention

(n = 75)

Received allocated intervention (n = …)

Did not receive allocated intervention (n = …)

(give reasons)

Allocated to Individualised intervention

(n = 75)Received allocated intervention (n = …)

Did not receive allocated intervention (n = …)

(give reasons)

**Allocation**

**Follow up**

Lost to follow up

(n = …) (give reasons)Discontinued intervention (n = …) (give reasons)

Lost to follow up

(n = …) (give reasons)Discontinued intervention (n = …) (give reasons)

# Analysis

Analyzed (n = …)

Excluded from analysis

(n = …) (give reasons)

Analyzed (n = …)

Excluded from analysis

(n = …) (give reasons)

FIGURE 1: Consort flow Chart

## 2.2 Registration and ethics

This study will be registered in Clinical Trials Registry- India (CTRI). The study protocol will be reviewed and approved by the institutional ethical committee of JSS Academy of Higher Education and Research, Mysuru, India. The Consolidated Standards of Reporting Trials (CONSORT) guidelines will be followed to report the study. Consent of the participant will be obtained before the commencement of the study.

## 2.3 Setting

The study will be conducted at Physiotherapy Outpatient Department (OPD) of JSS Hospital, Mysuru, India and the associated community centers affiliated to JSS Hospital.

## 2.4 Recruitment strategy

Patients with LBP visiting Physiotherapy OPD of JSS Hospital, Mysuru, India and patients who are part of the community outreach programs of JSS College of Physiotherapy in Mysuru, will be invited to participate in this study. The convenience sampling will be followed for the recruitment. Individuals who express interest in the study will be given detailed information regarding their involvement and the specifics of the study.

## 2.5 Sample size estimation

Sample size was calculated using G power using input parameters such as an effect size of 0.5, and power of 0.95. A sample size of 154 was received as an output from G power, allowing for loss to followup.

## 2.6 Participants

The initial selection criteria outlined in Table 1. Eligible patients will be informed about the study and will be invited to participate by signing a written informed consent before being enrolled.

| **Inclusion criteria** |
| --- |
| 1. A primary complaint of either:   1. low back pain, defined as pain between the inferior costal margin and the inferior gluteal fold with or without referral into the leg(s),   or   1. referred leg pain, defined as predominately unilateral posterior leg pain extending below the knee, or anterior thigh pain, with or without back pain (disc herniation with associated radiculopathy subgroup only)   2. Duration of the current episode of primary complaint lasting for greater than 3 months (chronic stage of the injury)  3. Aged between 18 and 65 (inclusive)  4. Fluency in English sufficient to complete questionnaires and to enable understanding of the intervention  5. Agreeing to refrain from other interventions wherever possible for the 10-week treatment period of the trial, aside from consultations with medical practitioners, medication, and any exercises already being undertaken. |
| **Exclusion criteria** |
| 1. Active cancer under current treatment, as the treatment of cancer may interfere with their ability to participate in the trial  2. Signs of cauda equina syndrome based on bladder or bowel disturbance and/or imaging [52]  3. Current pregnancy, or childbirth within the last 6 months, as this could impair the ability to undertake exercises, and could also cause back and leg symptoms that are not related to the subgroups under investigation  4. Spinal injections within the last 6 weeks, as we wish to study treatment effects independent of the effects of injections  5. Any history of lumbar spine surgery, as there is already considerable research evaluating the efficacy of post-surgical rehabilitation programs  6. A pain intensity score of less than 2/10 on a 0-10 numerical rating scale due to low severity  7. Minimal activity limitation, evidenced by a baseline ability to walk, sit, and stand for one hour or more and no sleep disturbance at night, as we wish to exclude people with low severity  8. Already received more than 5 sessions of physiotherapy with any of the treating physiotherapists before enrolment, as these therapists are likely to use many components of the trial treatment protocol on their usual client caseload  9. Inability to walk safely, such as severe foot drop causing regular tripping, as the interventions in the trial include walking for most participants  10. Planned absence of more than one week during the treatment period (such as overseas holidays) |

**Table 1: Eligibility criteria common to all subgroups**

## 2.7 Therapist

Registered physiotherapists in India, who are currently involved in LBP management, and undergone a STOPS training course led by the original Australian developers (Jon Ford, and Andrew Hahne).

## 2.8 Baseline evaluation

In addition to demographic and general health information of the participant, all the outcome measures (Table 2) provided below will be evaluated at baseline for both STOPS group as well as standard physiotherapy group. All the baseline assessment will be performed by a Physiotherapist who has a minimum of bachelor level degree and blinded allocation of the groups.

| **Patients’ outcome measures** | |
| --- | --- |
| **Outcome measures** | **Measurement points (**weeks) |
| **Primary outcome measures** | |
| 1. Oswestry Disability Index V2.1 with “sex life” question replaced by a “work/housework” question | 0, 5, 10, 26 and 52 |
| 2.Back pain and leg pain intensity :NPRS | 0, 5, 10, 26 and 52 |
|  |  |
| 3. Resting state functional MRI | 0,26 |
| **Secondary outcome measures** | |
| 1. Brief Pain Inventory | 0, 5, 10, 26 |
| 1. Global rating of change scale (7-point Likert scale) | 5, 10, 26 |
| 1. Satisfaction with treatment (5-point Likert scale) | 5, 10, 26 |
| 1. Interference with work or housework in the past week (5-point Likert scale) and number of hours missed at work in past week. | 0, 5, 10, 26 |
| 1. Örebro Musculoskeletal Pain Screening Questionnaire (ÖMPSQ-SF) | 0, 5, 10, 26 |
| 1. Quality of life (EuroQol-5D-5L) | 0, 5, 10, 26 |
| 1. Depression Anxiety and Stress Scale (DASS-21) | 0, 5, 10, 26 |
| 1. Pain Self-Efficacy questionnaire (PSEQ) | 0, 5, 10, 26 |
| 1. Pain Catastrophizing Scale (PCS) | 0, 5, 10, 26 |
| 1. Insomnia Severity Index (ISI) | 0, 5, 10, 26 |
| 1. Central Sensitisation Inventory (CSI) | 0, 5, 10, 26 |
| 1. Clinical inflammation score | 0, 5, 10, 26 |
| 1. Treatment Credibility Questionnaire | 5, 10, 26 |
| 1. Healthcare utilisation | 5, 10, 26 |
| 1. Compliance with treatment | 5, 10, 26, 52 |
| 1. Resting state functional MRI | 0, 26 |
| 1. Qualitative interviews | At the end of treatment trial 10 |
| 1. Adverse event | 5, 10, 26 |

**Table 2: Patient outcome measures**

## 2.9 Randomization and allocation concealment

Eligible participants will be randomized in to STOPS group or standard physiotherapy group using web based open source randomization software, by a researcher who is not involved in any other phase of the study (participant screening or enrolment).

To ensure an equal number of participants in both groups and reduce allocation bias, the allocation will be concealed through block randomization. The administrator will create sealed envelopes with allocation information, which will be given to participants by the assessor after the baseline assessment is completed. Both patients and outcome evaluators will be blinded to group allocation.

## 2.10 Interventions

### 2.10.1 STOPS group

***Classification of patients into subgroups***

Those found to be eligible will be invited to attend individualized assessment with trained physiotherapists. Information from the initial eligibility criteria and physical examination (Appendix 1) will be used to confirm eligibility, determine which subgroup (if any) the patient fits and provide descriptive information on the baseline characteristics of the patients. The patients will be classified into one of the five pre-defined subgroups; i) disc herniation with associated radiculopathy, ii) reducible discogenic pain, iii) non-reducible discogenic pain, iv) zygapophyseal joint dysfunction, v) Sacroiliac dysfunction vi) Non identifiable pathoanatomical disorder, or vii) others subgroup based on information from the eligibility criteria and physical examination. Additionally, patients in all STOPS subgroups will also be further evaluated according to their dominant pain type (nociplastic, neuropathic or nociceptive) using validated assessment. The detailed justification for each of the subgroups has been reported in the previous STOPS classification and treatment protocols.^[14,56-63]^ Additionally, an overview and description of the subgroups is reported in Appendix 2.

**Neuropathic**

**Nociceptive**

**Nociplastic**

**ZJD**

**Other**

**SIJ**

**Inflammation**

**RDP**

**NRDP**

**Radicular disorders**

No identifiable patho-**anatomical** diagnosis

**Depression**

**Anxiety**

**Stress**

**Catastrophizing**

**Pain Self Efficacy**

**Unhelpful motor control strategies**

**Sleep Dysfunction**

**Work issues**

**Unhelpful Beliefs**

**Co morbidities**

**Relationships**

**Readiness for change**

**PTSD**

**Stage 1:**

**Pain type**

**Stage 2:**

**Pathoanatomical diagnosis (if any)**

**Deconditioning**

**Stage 3:**

**Potential barriers to recovery**

**Figure 2: STOPS decision rule algorithm for classifying participants into subgroups**

**STOPS treatment**

Patients will receive individualized physiotherapy sessions, specific to each subgroup, over 10 weeks. The protocols for each subgroup will be outlined in a detailed treatment manual, supplemented by clinical notes which will contain an outline of the treatment protocol including a series of decision-making algorithms on a session-by-session basis. The algorithms (Figure 2) and clinical notes will ensure that essential elements of the treatment program are consistently applied by all physiotherapists across all patients, while still allowing some opportunity for the treatment to be tailored to individual patients. The clinical notes will also require the physiotherapists to document assessment or reassessment findings, clinical reasoning or decision-making rationale, treatment provided and response to treatment during each session. A detailed description (Appendix 2) of the treatment methods, clinical notes and decision-making algorithms for each subgroup will be based broadly on the principles outlined in the Specific Treatment of Problems of the Spine (STOPS) trial treatment protocols. ^[14,56-63]^ A summary of the treatment components is outlined in Table 3.

### 2.10.2 Usual care group

Participants in the usual care receive the routine physical therapy regimen of 10 sessions over 10 weeks

| **Intervention description:** | | | |
| --- | --- | --- | --- |
|  | STOPS physiotherapy | Usual physiotherapy care |  |
| Brief name | STOPS | Usual care |  |
| Why | Improve pain and activity limitation by addressing each participant’s individual barriers to recovery, with better maintenance of treatment effects in the long-term | Short-term relief of pain and function through traditional treatment methods that meet the expectations of participants |  |
| What materials | STOPS treatment protocols.  Decision-making flowcharts.  Treatment based on thorough assessment and questionnaire battery. | Usual care treatment as per protocols followed in Physiotherapy Outpatient Department (OPD) of JSS Hospital, Mysuru, India. |  |
| What procedures | In accordance with published STOPS protocols, modified for persistent pain. | Usual treatment procedures at discretion of physiotherapist |  |
| Who provided | Physiotherapist who has received training in the STOPS approach for managing persistent low back pain | Physiotherapists unfamiliar with the STOPS approach |  |
| How provided | Individual face-to-face sessions | Individual face-to-face sessions |  |
| Where (setting) | Participant with chronic LBP living independently in the community. Treatment provided at Federal Physiotherapy Outpatient Department (OPD) of JSS Hospital, Mysuru, India and the associated community centers affiliated to JSS Hospital. | Participant with chronic LBP living independently in the community. Physiotherapy Outpatient Department (OPD) of JSS Hospital, Mysuru, India and the associated community centers affiliated to JSS Hospital. |  |
| When/how much (dose) | 11 x 45-minute sessions (10sessions in 10 weeks) and the 11^th^ at 6-months post trail commencement | 11 x 45-minute sessions (10 sessions in the 10 weeks) and the 11^th^ at 6-months post trail commencement |  |
| Tailoring | Sessions tailored to the needs and progress of the individual in accordance with the STOPS protocols and detailed assessment profile of the participant | Sessions tailored to the needs and progress of the individual based on standard procedures used by the physiotherapist |  |
| Fidelity checking measures | Physiotherapists must pass a written and practical examination prior to commencing treatment in the trial  A minimum of one treatment session per month will be video recorded and assessed by the researchers for compliance with treatment protocols  Individual patient notes will be reviewed twice throughout the treatment program for each participant by the researchers, to evaluate fidelity and to provide timely feedback | Treatment will be recorded to determine its contents. |  |

**Table 3: Description of experimental and comparison group treatments according to the template for intervention description and replication (TIDieR)**

## 2.11 Treatment fidelity

The Physiotherapists treating the intervention group will undergo a three-month training program in Australia, where they will participate in training and observation sessions with researchers from La Trobe University and Advance Healthcare. These activities will include:

- Observation of Australian physiotherapists trained in the STOPS approach as they assess and manage people with LBP using the STOPS protocols
- Tutorials and peer learning involving Australian physiotherapists and other interns
- Preparation and presentation of case studies
- Intensive study of the STOPS protocols
- Completion of written and practical examinations to ensure sufficient mastery of the protocols to serve the role of the primary clinical contact in India.

After commencing the trial, physiotherapists will be monitored for the quality of their treatment via the following process:

- Regular review of treatment notes by the original STOPS developers (Andrew Hahne and Jon Ford).
- If the competency and proficiency threshold of “fair” is not met, further top-up training for the physiotherapists will be implemented followed by re-evaluation
- If physiotherapists were not engaged in providing treatment in the trial for longer than 12 weeks, top-up training and re-evaluation will also be implemented.

## 2.12 Outcome measures

A research assistant will be available to assist patients with the questionnaires as needed and to collect them upon completion.

***Primary outcomes***

Activity limitation will be evaluated using nine questions from the Oswestry Disability Index Version 2.1^[66,67]^, with the tenth question relating to “sex life” being replaced by a question relating to “work/housework”.^[67,68]^ The Oswestry is a reliable, valid, and responsive instrument for measuring activity limitation in people with low back pain and referred leg pain.^[69-71]^

***Secondary outcomes***

1. *Back pain and leg pain intensity:* Separate 0-10 Numerical Rating Pain Scales will be administered, one for back pain and another for leg pain. The intensity of pain will be rated on average over the past week, with end-point descriptors of “no pain” and “worst pain possible”.
2. *Pain severity and impact:* Brief Pain Inventory - Short Form (BPI-SF) will be used to assess the severity of pain and the impact of pain on daily functions. The BPI-SF is a 9-item self-administered questionnaire which exists within the [biopsychosocial model](https://www.physio-pedia.com/Biopsychosocial_Model) of pain, as it addresses sensory, emotional, and functional aspects of the pain experience ^[72]^. Thus, the tool is responsive to changes in pain associated with both pharmacological, physical, and psychological interventions^[73]^
3. *Global effect:* Global rating of change will be measured using a 7-point Likert scale, with participants rating their overall change since the baseline assessment as “completely recovered”, “much improved”, “slightly improved”, “no change”, “slightly worsened”, “much worsened”, or “vastly worsened”.^[74-75]^ Various versions of this scale are reliable, responsive, and valid. ^[75-76]^
4. *Satisfaction with treatment:* Participants will rate their satisfaction with physiotherapy treatment on a 5-point Likert scale, with ratings from “very satisfied” to “very dissatisfied”.^[77,78]^This scale has good reliability, validity, and responsiveness. ^[79,80]^
5. *Work interference:* Interference with work due to the LBP will be assessed in two ways. Firstly, at each assessment point, participants will record the number of workdays missed due to their back/leg condition over the previous 30 days. ^[14,56]^ Secondly, participants will rate the degree of interference with work (employment or housework) caused by their back/leg condition over the previous week on a five-point scale ranging from “not at all” to “extremely”. ^[14,56]^ These measurement methods have demonstrated good reliability, validity, and responsiveness ^[81,82]^.
6. *Psychosocial risk factors:* The Short-Form Örebro Musculoskeletal Pain Screening Questionnaire (ÖMPSQ-SF) will be used as a measure of psychosocial risk factors for chronic musculoskeletal pain ^[83,84]^. Although it is more commonly used as a prognostic screening tool at one point in time ^[85]^, in our trial the Orebro will also be administered at each follow-up point to detect changes in psychosocial risk factors over time. This appears justified given that Orebro has good test-retest reliability and internal consistency ^[83,86]^.
7. *Health-related quality of life:* Health-related quality of life will be measured with the EuoQol-5D-5L ^[87]^. Utilities will be calculated according to the validated algorithms of Dolan ^[88]^. The EuroQol-5D-5L has good reliability, validity, and ^responsiveness [89-91]^, and has been used in other low back pain trials ^[64,92]^.
8. *Mental health symptoms:* Mental health symptoms will be assessed using the 21-item Depression Anxiety and Stress Scale (DASS-21). The questionnaire measures the severity of depression, anxiety, and stress from normal to extremely severe ^[93]^. DASS-21 normal score cut-offs are 9 for depression, 7 for anxiety, and 14 for stress and higher scores suggest mild to extremely severe mental health symptoms ^[93]^. DASS-21 shows strong validity, test-retest reliability, internal consistency, and responsiveness ^[93,94]^.
9. *Confidence with activities:* Confidence with activities of daily living despite having pain will be assessed using the Pain Self-Efficacy Questionnaire (PSEQ). The PSEQ is a 10-item scale with a score ranging from 0 to 60, where high scores indicate greater levels of confidence in dealing with pain ^[95]^. The PSEQ has excellent validity, reliability, and responsiveness ^[96]^.
10. *Pain catastrophising:* The 13-item Pain Catastrophizing Scale (PCS) will be used to assess inappropriate coping strategies and catastrophic thinking about LBP. The scale is rated on a 5-point Likert scale ranging from 0 (not at all) to 4 (all the time) ^[97]^. Total scores range from 0 to 52, with higher scores indicating worse catastrophic thinking about LBP ^[97]^. The PCS has been shown to have adequate validity, reliability and responsiveness ^[97,98]^.
11. *Sleep quality:* The Insomnia Severity Index (ISI) will be used to assess sleep quality. The scale comprises 7 questions that assess current sleep problems that are rated on a 5-point Likert scale (‘0’ representing none or not at all and ‘4’ representing very much) ^[99]^. Total scores range from 0 to 28, with higher scores indicating worse insomnia severity ^[99]^. The ISI demonstrated good validity ^[99]^ and responsiveness ^[100]^ and has been used to assess sleep quality in individuals with LBP ^[101]^.
12. *Central sensitisation:* The Central Sensitisation Inventory (CSI) will be used to assess central sensitization symptoms. The CSI is a 25-point scale, rated on a scale of 0 (never) to 4 (always), with a score of more than 40 indicating the presence of central sensitization ^[102]^. The CSI is a useful and valid measure for screening patients with central sensitization symptoms ^[103]^ and has strong psychometric properties ^[104, 105]^
13. *Inflammatory symptoms:* The 4-item clinical inflammation score will be used to assess low back-related inflammatory symptoms. A positive clinical inflammation score of at least 3 of; constant symptoms, morning pain/stiffness greater than 60-min, short walking not easing symptoms and significant night symptoms will be used to inform clinical decision. This scale has been validated and achieved a sensitivity of 90.9%, a specificity of 92.9%, and predictive accuracy of 92.3% ^[106]^.
14. *Treatment acceptability:* The treatment Credibility Questionnaire will be used to determine the acceptability of each treatment approach ^[107]^. Treatment acceptability will be assessed at each follow-up visit via a 4-item questionnaire rated on a scale of 0-10, at this point, how logical does the treatment offered to you seem? At this point, how successful do you think this treatment will be in helping you with your back problem? How confident would you be in recommending this treatment to a friend who experiences similar problems? By the end of your treatment, how much improvement in your back condition do you think will occur? This questionnaire demonstrated high internal consistency and good test-retest reliability ^[107]^ and has been used in LBP trials ^[108, 109]^.
15. *Healthcare utilization:* A patient diary will be used to track healthcare utilization including imaging, medication, and other healthcare services ^[14,56]^.
16. *Compliance with treatment:* The number of treatment sessions attended, and the number of missed or cancelled appointments by each patient will be recorded by the physiotherapists. Patient compliance with the treatment will be reviewed by the physiotherapists at each follow-up visit via direct questioning (on a scale of 0-10, how adherent have you been with following the advice and exercises recommended by your physiotherapist on average over the last month? from “not at all” to “fully”) and by reviewing the patient’s exercise charts ^[14,56]^.
17. Resting state functional MRI data (only for a cohort).Details under (Appendix 4)
18. *Qualitative interviews:* A qualitative study of therapist and patients’ experiences will be nested within the usual care program and quality improvement programme using a semi-structured questionnaire and interviews. Individual interviews lasting for 30-40 minutes will be conducted by a different researcher who has experience in qualitative interviews but was not involved in treating patients in the study. Interviews will be voice recorded, and transcribed verbatim, before undergoing thematic analysis. All discussions will be directed by a flexible topic guide (Appendix 3) developed from the pertinent literature ^[110-113]^. The topic guide comprises semi-structured non-leading questions phrased in clear language.
19. *Adverse events:* Continuous clinical assessment of adverse effects events (harmful or unpleasant) of treatment will be monitored by treating physiotherapists and recorded in their participant clinical notes, per their standard clinical practice. Physiotherapists will routinely enquire with participants each session about their response to treatment and any adverse effects of treatment or prescribed exercises.

## 2.13 Follow-up

Patients will be assessed using self-administered questionnaires that will be filled in physically at the clinic at baseline, at the 5-week and 10-week treatment sessions, and then at the 26- weeks and 52-weeks (for only primary outcomes: disability) post-enrolment.

## 2.14 Compliance with treatment

A research assistant who is a staff member in department of physiotherapy, JSSCPT and not involved in low back pain treatment will assist participants to complete their questionnaires, which include self-reported measures of compliance with treatment. Day-to-day compliance with exercises will be monitored by the treating physiotherapists as part of their usual practice.

## 2.15 Data analysis

Data will be analyzed using IBM SPSS-V23.0 (SPSS Inc., Chicago, Illinois, USA) and Microsoft Excel^®^ (2010; Microsoft Corporation, Redmond, WA, USA).

***Analysis of patients’ clinical outcomes***

A descriptive summary of the characteristics of the patients will be provided at baseline. Continuous outcomes for patients will be analysed using linear mixed models (group × time interaction, with time modelled as a repeated measure) adjusting for baseline score. Ordinal outcomes will be analysed using the Mann-Whitney U test, while the risk ratio and number needed to treat will be evaluated using χ2 analyses. Analyses will focus on detecting between-group effects (with 95% CIs) at each time point (5, 10, and 26 weeks), and participants will be dichotomized according to whether they achieve the minimal clinically important difference (MCID) on outcome measures or not. The MCID for participants will be defined as 10/100 points for the Oswestry, 2/10 for the BPI pain scales, at least ‘much improved’ on the global rating of change scale, and ‘very satisfied’ on the treatment satisfaction scales. Given that these values may be too low in some contexts, the analyses will be repeated using a threshold of 50% reduction in Oswestry and NRS pain scores [14,56]. All data will be analysed according to intention-to-treat analysis (ITT), with missing data handled via maximum likelihood estimation ^[114]^.

***Analysis of cost-effectiveness***

Health care costs including prescribed treatment and any co-interventions for each participant will be recorded from the date of the first enrolment to the 6-month follow-up. Healthcare resource utilisation will be obtained using standardised follow-up questionnaires mailed to participants at baseline, 5, 10, and 26-week post-enrolment. A within-study cost-utility analysis will be conducted from the health care perspective and all costs will be valued based on TIER 2 city charges ( Mysuru under Tier 2 city as per the government of India)and will be reported in Rupees.

All analyses will be via intention to treat, with imputation used for missing data. First, partially missing information relating to healthcare resource utilization will be replaced with relevant averages (e.g., if a patient indicated that they consumed Voltaren but did not indicate the dosage, then the average dose will be imputed from all other patients in that treatment group at that time point). Fully missing data (such as patients who failed to return a questionnaire, missed an item of the EuroQol, or failed to answer a complete question) will be handled via multiple imputations (five imputed data sets).^[115-116]^

Between-group differences in costs will be calculated via linear mixed models. For health outcomes, each patient’s time-weighted average EuroQol utility score across the 6-month study period will be calculated via the area under the curve method ^[117]^. This will yield health outcomes expressed in quality-adjusted life years (QALYs), which indicates the cost to gain one additional year of life spent in perfect health ^[118]^. The mean between-group difference in QALYs will then be derived from a linear mixed model adjusting for baseline scores, using the multiplied imputed data sets. The mean between-group differences in health care and work costs will also be derived from linear mixed models based on the imputed data sets. These analyses will yield incremental costs and incremental health benefits. The ICER will then be derived by dividing the incremental cost by the incremental QALYs, which indicates the cost to gain one additional QALY. ^[115,118]^

To assess uncertainty around the ICER, nonparametric bootstrapping will be applied using a customized Microsoft Excel spreadsheet. ^[119]^ This will involve generating 5000 randomly resampled data sets (1000 samples for each of the five imputed data sets). Each of the 5000 bootstrapped cost-utility pairs will then be graphed on the cost-effectiveness plane ^[118,120,121]^. A cost-effectiveness acceptability curve will then be generated to determine the probability that the STOPS treatment approach for LBP is cost-effective compared with usual.

***Analysis of qualitative data***

The qualitative data will be analyzed using content analysis^[122]^. The interview documents will be read several times by the investigators to understand the texts and the meaning of the content according to the local context. In the next step, the documents will be combined into meaningful and understandable units which will be labelled as codes. The coding process will be performed by two investigators separately, and then compared, and discussed and consensus will be reached within the research team. In the third step, the codes will be synthesized in detail and will be grouped into meaningful sub-categories and labelled with appropriate titles. In the fourth step, subcategories will be merged into broader categories which will finally be pooled into major emerging themes.

***Analysis of fMRI data***

Data will be preprocessed and analyzed using SPM12 (the welcome department of cognitive neurology, London, UK, <http://www.fil.ion.ucl.ac.uk/spm/software/spm12/>). The preprocessing pipeline used will be the standard conventional preprocessing pipeline in using the CONN-fMRI functional connectivity toolbox (http://www.nitrc.org/projects/conn).

All functional images will be slice-time corrected and realigned to the first volume using a six-parameter rigid body transformation. The anatomical image and functional images will be coregistered for the corresponding time-point. Segmented gray matter and white matter images of all participants will be used to construct a tissue probability maps. The template will be normalized to Montreal Neurological Institute (MNI) space and all images, anatomical and functional, will be normalized to this template using the according flow fields. The smoothing kernel for the functional images will be kept as 6 mm and 2 mm for the anatomical image. 2.5.

Functional connectivity analyses will be carried out using the CONN-fMRI functional connectivity toolbox v14. Seed-to-voxel and ROI-to-ROI functional connectivity maps will be created for each participant. The ROI-to-ROI analysis will be used to identify possible differences between before and after treatment. For this analysis we will use all the provided areas. The mean BOLD time series will be computed across all voxels within each ROI.

Individual seed-to-voxel and ROI-to-ROI maps will be entered into a second-level analysis. A within group ROI-to-ROI analysis will be performed. Seed-to-voxel analyses will be as necessary.

## Reference:

1. Wang L, Ye H, Li Z, Lu C, Ye J, Liao M, Chen X. Epidemiological trends of low back pain at the global, regional, and national levels. European Spine Journal. 2022 Apr;31(4):953-62.
2. Vos T, Lim SS, Abbafati C, Abbas KM, Abbasi M, Abbasifard M, Abbasi-Kangevari M, Abbastabar H, Abd-Allah F, Abdelalim A, Abdollahi M. Global burden of 369 diseases and injuries in 204 countries and territories, 1990–2019: a systematic analysis for the Global Burden of Disease Study 2019. The Lancet. 2020 Oct 17;396(10258):1204-22.
3. Wu A, March L, Zheng X, Huang J, Wang X, Zhao J, Blyth FM, Smith E, Buchbinder R, Hoy D. Global low back pain prevalence and years lived with disability from 1990 to 2017: estimates from the Global Burden of Disease Study 2017. Annals of translational medicine.2020 Mar;8(6).
4. ICMR P. IHME (2017) India: health of the nation’s states-the India state-level disease burden initiative. Indian Council of Medical Research. Public Health Foundation of India, Institute for Health Metrics and Evaluation, New Delhi. 2017
5. Global Burden of Disease study 2019: a systematic analysis for the Global Burden of Disease Study 2019,The Lancet, Volume 396, Issue 10267, 2020, Pages 2006-2017
6. J Hartvigsen, MJ Hancock, A Kongsted, *et al.* What low back pain is and why we need to pay attention Lancet, 391 (2018), pp. 2356-2367
7. Hodges PW. Hybrid Approach to Treatment Tailoring for Low Back Pain: A Proposed Model of Care. J Orthop Sports Phys Ther. 2019 Jun;49(6):453-463. doi: 10.2519/jospt.2019.8774
8. Mescouto K, Olson RE, Hodges PW, Setchell J. A critical review of the biopsychosocial model of low back pain care: time for a new approach? Disabil Rehabil. 2020 Dec 7:1-15. doi: 10.1080/09638288.2020.1851783
9. O'Connell NE, Cook CE, Wand BM, Ward SP. Clinical guidelines for low back pain: a critical review of consensus and inconsistencies across three major guidelines. Best practice & research Clinical rheumatology. 2016 Dec 1;30(6):968-80.
10. George SZ, Fritz JM, Silfies SP, Schneider MJ, Beneciuk JM, Lentz TA, Gilliam JR, Hendren S, Norman KS, Beattie PF, Bishop MD. Interventions for the management of acute and chronic low back pain: revision 2021: clinical practice guidelines linked to the international classification of functioning, disability and health from the academy of orthopaedic physical therapy of the American Physical Therapy Association. Journal of Orthopaedic & Sports Physical Therapy. 2021 Nov;51(11):CPG1-60.
11. Corp N, Mansell G, Stynes S, Wynne-Jones G, Morsø L, Hill JC, van der Windt DA. Evidence-based treatment recommendations for neck and low back pain across Europe: A systematic review of guidelines. Eur J Pain. 2021 Feb;25(2):275-295. doi: 10.1002/ejp.1679. Epub 2020 Nov 12. PMID: 33064878; PMCID: PMC7839780.
12. Hill JC, Whitehurst DG, Lewis M, Bryan S, Dunn KM, Foster NE, Konstantinou K, Main CJ, Mason E, Somerville S, Sowden G, Vohora K, Hay EM. Comparison of stratified primary care management for low back pain with current best practice (STarT Back): a randomised controlled trial. Lancet. 2011 Oct 29;378(9802):1560-71. doi: 10.1016/S0140-6736(11)60937-9
13. Vibe Fersum K, O'Sullivan P, Skouen JS, Smith A, Kvale A. Efficacy of classification-based cognitive functional therapy in patients with non-specific chronic low back pain: a randomized controlled trial. *Eur J Pain*. 2013;17:916-928.
14. Ford JJ, Hahne AJ, Surkitt LD, Chan AY, Richards MC, Slater SL, Hinman RS, Pizzari T, Davidson M, Taylor NF. Individualised physiotherapy as an adjunct to guideline-based advice for low back disorders in primary care: a randomised controlled trial. Br J Sports Med. 2016 Feb;50(4):237-45. doi: 10.1136/bjsports-2015-095058
15. Lehtola V, Luomajoki H, Leinonen V, Gibbons S, Airaksinen O. Sub-classification based specific movement control exercises are superior to general exercise in sub-acute low back pain when both are combined with manual therapy: A randomized controlled trial. BMC Musculoskelet Disord. 2016 Mar 22;17:135. doi: 10.1186/s12891-016-0986
16. O'Keeffe M, O'Sullivan P, Purtill H, Bargary N, O'Sullivan K. Cognitive functional therapy compared with a group-based exercise and education intervention for chronic low back pain: a multicentre randomised controlled trial (RCT). Br J Sports Med. 2020 Jul;54(13):782-789. doi: 10.1136/bjsports-2019-100780. Epub 2019 Oct 19. PMID: 31630089; PMCID: PMC7361017.
17. Karin Schröder, Birgitta Öberg, Paul Enthoven, Henrik Hedevik & Allan Abbott (2022): Improved adherence to clinical guidelines for low back pain after implementation of the BetterBack model of care: A stepped cluster randomized controlled trial within a hybrid type 2 trial, Physiotherapy Theory and Practice, DOI: 10.1080/09593985.2022.2040669
18. Foster NE, Anema JR, Cherkin D, Chou R, Cohen SP, Gross DP, et al.; Lancet Low Back Pain Series Working Group. Prevention and treatment of low back pain: evidence, challenges, and promising directions. Lancet. 2018 06 9;391(10137):2368–83. doi: <http://dx.doi.org/10.1016/S0140-6736(18)30489-6>
19. Buchbinder R, van Tulder M, Öberg B, Costa LM, Woolf A, Schoene M, et al.; Lancet Low Back Pain Series Working Group. Low back pain: a call for action. Lancet. 2018 06 9;391(10137):2384–8. doi: <http://dx.doi.org/10.1016/S0140-6736(18)30488-4>
20. Fidvi N, May S. Physiotherapy management of low back pain in India - a survey of self-reported practice. Physiother Res Int. 2010 Sep;15(3):150-9. doi: 10.1002/pri.458. PMID: 20108238.
21. Zadro J, O’Keeffe M,Maher C. Do physical therapists follow evidence-based guidelines when managing musculoskeletal conditions? Systematic review. BMJ Open 2019;9:e032329. doi:10.1136/ bmjopen-2019-032329.
22. Gardner, T., Refshauge, K., Smith, L., McAuley, J., Hubscher, M., Goodall, S., 2017.Physiotherapists' beliefs and attitudes influence clinical practice in chronic low backpain: a systematic review of quantitative and qualitative studies. J. Physiother. 63, 132–143.
23. Christe G, Nzamba J, Desarzens L, Leuba A, Darlow B, Pichonnaz C. Physiotherapists’ attitudes and beliefs about low back pain influence their clinical decisions and advice. Musculoskeletal science and practice. 2021 Jun 1;53:102382.
24. Hong JY, Song KS, Cho JH, Lee JH, Kim NH. An Updated Overview of Low Back Pain Management. Asian Spine J. 2021 Dec 30. doi: 10.31616/asj.2021.0371
25. Kreiner DS, Matz P, Bono CM, et al. Guideline summary review: an evidence-based clinical guideline for the diagnosis and treatment of low back pain. Spine J. 2020 Jul;20(7):998-1024. doi: 10.1016/j.spinee.2020.04.006
26. Hong JY, Song KS, Cho JH, Lee JH. An Updated Overview of Low Back Pain Management in Primary Care. Asian Spine J. 2017 Aug;11(4):653-660. doi: 10.4184/asj.2017.11.4.653
27. Chenot JF, Greitemann B, Kladny B, et al. Nonspecific low back pain. Dtsch Arztebl Int. 2017 12 25;114(51-52):883–90.
28. Management of people with acute low back pain model of care. Chatswood: New South Wales Agency for Clinical Innovation; 2016. Available from: https://www.aci.health.nsw.gov.au/__data/assets/pdf_file/0007/336688/acute-low-back-pain-moc.pdf [cited 2019 Feb 15].
29. Qaseem A, Wilt TJ, McLean RM, Forciea MA. Clinical Guidelines Committee of the American College of Physicians. Noninvasive treatments for acute, subacute, and chronic low back pain: a clinical practice guideline from the American College of Physicians. Ann Intern Med. 2017 Apr 4;166(7):514–30. doi: <http://dx.doi.org/10.7326/M16-2367>
30. Stochkendahl MJ, Kjaer P, Hartvigsen J, Kongsted A, Aaboe J, Andersen M, et al. National clinical guidelines for non-surgical treatment of patients with recent onset low back pain or lumbar radiculopathy. Eur Spine J. 2018 01;27(1):60–75. doi: [10.1007/s00586-017-5099-2](http://dx.doi.org/10.1007/s00586-017-5099-2)
31. Corp N, Mansell G, Stynes S, Wynne‐Jones G, Morsø L, Hill JC, van der Windt DA. Evidence‐based treatment recommendations for neck and low back pain across Europe: a systematic review of guidelines. European Journal of Pain. 2021 Feb;25(2):275-95.,
32. Oliveira CB, Maher CG, Pinto RZ, Traeger AC, Lin CW, Chenot JF, van Tulder M, Koes BW. Clinical practice guidelines for the management of non-specific low back pain in primary care: an updated overview. European Spine Journal. 2018 Nov;27:2791-803.
33. Meroni R, Piscitelli D, Ravasio C, Vanti C, Bertozzi L, De Vito G, Perin C, Guccione AA, Cerri CG, Pillastrini P. Evidence for managing chronic low back pain in primary care: a review of recommendations from high-quality clinical practice guidelines. Disability and rehabilitation. 2021 Mar 27;43(7):1029-43.
34. Cohen SP, Bhaskar A, Bhatia A, Buvanendran A, Deer T, Garg S, Hooten WM, Hurley RW, Kennedy DJ, McLean BC, Moon JY. Consensus practice guidelines on interventions for lumbar facet joint pain from a multispecialty, international working group. Regional Anesthesia & Pain Medicine. 2020 Jun 1;45(6):424-67.
35. Longtin C, Decary S, Cook CE, Tousignant‐Laflamme Y. What does it take to facilitate the integration of clinical practice guidelines for the management of low back pain into practice? Part 1: a synthesis of recommendation. Pain Practice. 2021 Nov;21(8):943-54.
36. van Erp RMA, Huijnen IPJ, Jakobs MLG, Kleijnen J, Smeets RJEM. Effectiveness of Primary Care Interventions Using a Biopsychosocial Approach in Chronic Low Back Pain: A Systematic Review. Pain Pract. 2019 Feb;19(2):224-241. doi: 10.1111/papr.12735. Epub 2018 Dec 2. PMID: 30290052; PMCID: PMC7379915.
37. Rabey M, Smith A, Kent P, Beales D, Slater H, O’Sullivan P. Chronic low back pain is highly individualised: patterns of classification across three unidimensional subgrouping analyses. Scandinavian journal of pain. 2019 Oct 1;19(4):743-53.
38. Salathé CR, Melloh M, Crawford R, Scherrer S, Boos N, Elfering A. Treatment efficacy, clinical utility, and cost-effectiveness of multidisciplinary biopsychosocial rehabilitation treatments for persistent low back pain: a systematic review. Global spine journal. 2018 Dec;8(8):872-86.
39. Hefford C. McKenzie classification of mechanical spinal pain: profile of syndromes and directions of preference. *Man Ther.* 2008;13:75–81.
40. Petersen T, Laslett M, Thorsen H, Manniche C, Ekdahl C, Jacobsen S: Diagnostic classification of non-specific low back pain. A new system integrating patho-anatomic and clinical categories. Physiother Theory & Practice. 2003, 19: 213-237.
41. Sahrmann S, Azevedo DC, Dillen LV. Diagnosis and treatment of movement system impairment syndromes. *Braz J Phys Ther*. 2017;21(6):391-399. doi:10.1016/j.bjpt.2017.08.001
42. Karayannis, N.V., Jull, G.A. & Hodges, P.W. Physiotherapy movement-based classification approaches to low back pain: comparison of subgroups through review and developer/expert survey. *BMC Musculoskelet Disord* 13, 24 (2012). [10.1186/1471-2474-13-24](https://doi.org/10.1186/1471-2474-13-24)
43. Alrwaily M, Timko M, Schneider M, Stevans J, Bise C, Hariharan K, Delitto A. Treatment-Based Classification System for Low Back Pain: Revision and Update. Phys Ther. 2016 Jul;96(7):1057-66. doi: 10.2522/ptj.20150345. Epub 2015 Dec 4. PMID: 26637653
44. Tagliaferri SD, Mitchell UH, Saueressig T, Owen PJ, Miller CT, Belavy DL. Classification approaches for treating low back pain have small effects that are not clinically meaningful: A systematic review with meta-analysis. Journal of Orthopaedic & Sports Physical Therapy. 2022 Feb;52(2):67-84.
45. O’Kee_e, M.; Purtill, H.; Kennedy, N.; Conneely, M.; Hurley, J.; O’Sullivan, P.; Dankaerts,W.; O’Sullivan, K.Comparative E_ectiveness of Conservative Interventions for Nonspecific Chronic Spinal Pain: Physical, Behavioral/ Psychologically Informed, or Combined? A Systematic Review and Meta-Analysis. J. Pain **2016**, 17, 755–774
46. Han CS, Hancock MJ, Maher CG. Reconsidering non-specific low back pain: where to from here?. The Spine Journal. 2022 Dec 1;22(12):1927-30.
47. Ford J, Hahne A, Surkitt L, Chan A, Richards M. The evolving case supporting individualised physiotherapy for low back pain. Journal of clinical medicine. 2019 Aug 28;8(9):1334.
48. Longtin C, Decary S, Cook CE, Tousignant‐Laflamme Y. What does it take to facilitate the integration of clinical practice guidelines for the management of low back pain into practice? Part 1: a synthesis of recommendation. Pain Practice. 2021 Nov;21(8):943-54
49. Hancock MJ, Maher CG, Laslett M, Hay E, Koes B. Discussion paper: what happened to the ‘bio’ in the bio-psycho-social model of low back pain? Eur Spine J. 2011;20(12):2105–10.
50. Ford JJ, Hahne AJ. Pathoanatomy and classification of low back disorders. Man Ther. 2013;18:165–8.
51. Petersen, T., Laslett, M. & Juhl, C. Clinical classification in low back pain: best-evidence diagnostic rules based on systematic reviews. *BMC Musculoskelet Disord* 18, 188 (2017). <https://doi.org/10.1186/s12891-017-1549-6>
52. Cholewicki J, Breen A, Popovich JM Jr, Reeves NP, Sahrmann SA, van Dillen LR, Vleeming A, Hodges PW. Can Biomechanics Research Lead to More Effective Treatment of Low Back Pain? A Point-Counterpoint Debate. J Orthop Sports Phys Ther. 2019 Jun;49(6):425-436. doi: 10.2519/jospt.2019.8825
53. Roberto Meroni, Daniele Piscitelli, Claudio Ravasio, Carla Vanti, Lucia Bertozzi, Giovanni De Vito, Cecilia Perin, Andrew A. Guccione, Cesare G. Cerri & Paolo Pillastrini (2021) Evidence for managing chronic low back pain in primary care: a review of recommendations from high-quality clinical practice guidelines, Disability and Rehabilitation, 43:7, 1029-1043, DOI: 10.1080/09638288.2019.1645888
54. Lin I, Wiles L, Waller R, Goucke R, Nagree Y, Gibberd M, Straker L, Maher CG, O’Sullivan PP. What does best practice care for musculoskeletal pain look like? Eleven consistent recommendations from high-quality clinical practice guidelines: systematic review. British journal of sports medicine. 2020 Jan 1;54(2):79-86.
55. Corp N, Mansell G, Stynes S, Wynne‐Jones G, Morsø L, Hill JC, van der Windt DA. Evidence‐based treatment recommendations for neck and low back pain across Europe: a systematic review of guidelines. European Journal of Pain. 2021 Feb;25(2):275-95.
56. Hahne AJ, Ford JJ, Surkitt LD, Richards MC, Chan AY, Thompson SL, et al. Specific treatment of problems of the spine (STOPS): design of a randomised controlled trial comparing specific physiotherapy versus advice for people with subacute low back disorders. BMC Musculoskelet Disord. 2011;12:104.
57. Ford JJ, Thompson SL, Hahne AJ. A classification and treatment protocol for low back disorders. Part 1—specific manual therapy. Phys Ther Rev 2011;16:168–77.
58. Ford JJ, Surkitt LD, Hahne AJ. A classification and treatment protocol for low back disorders. Part 2: directional preference management for reducible discogenic pain. Phys Ther Rev 2011;16:423–37.
59. Ford JJ, Hahne AJ, Chan AYP, et al. A classification and treatment protocol for low back disorders. Part 3: functional restoration for intervertebral disc related disorders. Phys Ther Rev 2012;17:55–75.
60. Ford JJ, Richards MJ, Hahne AJ. A classification and treatment protocol for low back disorders. Part 4: functional restoration for low back disorders associated with multifactorial persistent pain. Phys Ther Rev 2012;17:322–34.
61. Surkitt LD, Ford JJ, Chan AY, Richards MC, Slater SL, Pizzari T, Hahne AJ. Effects of individualised directional preference management versus advice for reducible discogenic pain: A pre-planned secondary analysis of a randomised controlled trial. Man Ther. 2016 Sep;25:69-80. doi: 10.1016/j.math.2016.06.002
62. Chan AYP, Ford JJ, Surkitt LD, Richards MC, Slater SL, Davidson M, Hahne AJ. Individualised functional restoration plus guideline-based advice vs advice alone for non-reducible discogenic low back pain: a randomised controlled trial. Physiotherapy. 2017 Jun;103(2):121-130. doi: 10.1016/j.physio.2016.08.001
63. Ford JJ, Slater SL, Richards MC, Surkitt LD, Chan AYP, Taylor NF, Hahne AJ. Individualised manual therapy plus guideline-based advice vs advice alone for people with clinical features of lumbar zygapophyseal joint pain: a randomised controlled trial. Physiotherapy. 2019 Mar;105(1):53-64. doi: 10.1016/j.physio.2018.07.008
64. Hahne AJ, Ford JJ, Surkitt LD, Richards MC, Chan AYP, Slater SL, Taylor NF. Individualized Physical Therapy Is Cost-Effective Compared With Guideline-Based Advice for People With Low Back Disorders. Spine (Phila Pa 1976). 2017 Feb;42(3):E169-E176. doi: 10.1097/BRS.0000000000001734
65. Liew BXW, Ford JJ, Scutari M, Hahne AJ (2021) How does individualised physiotherapy work for people with low back pain? A Bayesian Network analysis using randomised controlled trial data. PLoS ONE 16(10): e0258515. [doi: 10.1371/journal.pone.0258515](https://doi.org/10.1371/journal.pone.0258515).
66. Fairbank JC, Couper J, Davies JB, O’Brien JP: The Oswestry low back pain disability questionnaire. Physiotherapy 1980, 66(8):271-273.
67. Davidson M: Rasch analysis of three versions of the Oswestry Disability Questionnaire. Man Ther 2008, 13(3):221-231.
68. Fritz JM, Irrgang JJ: A comparison of a modified Oswestry Low Back Pain Disability Questionnaire and the Quebec Back Pain Disability Scale. Phys Ther 2001, 81(2):776-788.
69. Lauridsen HH, Hartvigsen J, Manniche C, Korsholm L, Grunnet-Nilsson N: Responsiveness and minimal clinically important difference for pain and disability instruments in low back pain patients. BMC Musculoskelet Disord 2006, 7:82.
70. Davidson M, Keating JL: A comparison of five low back disability questionnaires: reliability and responsiveness. Phys Ther 2002, 82(1):8-24.
71. Frost H, Lamb SE, Stewart-Brown S: Responsiveness of a patient specific outcome measure compared with the Oswestry Disability Index v2.1 and Roland and Morris Disability Questionnaire for patients with subacute and chronic low back pain. Spine 2008, 33(22):2450-2457.
72. Tan G, Jensen MP, Thornby JI, Shanti BF. Validation of the Brief Pain Inventory for chronic nonmalignant pain. J Pain. 2004 Mar;5(2):133-7. doi: 10.1016/j.jpain.2003.12.005
73. Mendoza T, Mayne T, Rublee D, Cleeland C. Reliability and validity of a modified Brief Pain Inventory short form in patients with osteoarthritis. Eur J Pain. 2006 May;10(4):353-61. doi: 10.1016/j.ejpain.2005.06.002
74. Beurskens AJ, de Vet HC, Koke AJ: Responsiveness of functional status in low back pain: a comparison of different instruments. Pain 1996, 65(1):71-76.
75. Kamper S: Global Rating of Change scales. Aust J Physiother 2009, 55(4):289.
76. Kamper SJ, Maher CG, Mackay G: Global rating of change scales: a review of strengths and weaknesses and considerations for design. J Man Manip Ther 2009, 17(3):163-170.
77. Bombardier C: Outcome assessments in the evaluation of treatment of spinal disorders: summary and general recommendations. Spine 2000, 25(24):3100-3103.
78. Hudak PL, Wright JG: The characteristics of patient satisfaction measures. Spine 2000, 25(24):3167-3177.
79. Ferrer M, Pellise F, Escudero O, Alvarez L, Pont A, Alonso J, Deyo R: Validation of a minimum outcome core set in the evaluation of patients with back pain. Spine 2006, 31(12):1372-1379.
80. Mannion AF, Elfering A, Staerkle R, Junge A, Grob D, Semmer NK, Jacobshagen N, Dvorak J, Boos N: Outcome assessment in low back pain: how low can you go? Eur Spine J 2005, 14(10):1014-1026
81. Weinstein JN, Lurie JD, Tosteson TD, Skinner JS, Hanscom B, Tosteson AN, Herkowitz H, Fischgrund J, Cammisa FP, Albert T, et al: Surgical vs nonoperative treatment for lumbar disk herniation: the Spine Patient Outcomes Research Trial (SPORT) observational cohort. Jama 2006, 296(20):2451-2459.
82. Peul WC, van Houwelingen HC, van den Hout WB, Brand R, Eekhof JAH, Tans JTJ, Thomeer RTWM, Koes BW, Leiden-The Hague Spine Intervention Prognostic Study G: Surgery versus prolonged conservative treatment for sciatica. N Engl J Med 2007, 356(22):2245-2256.
83. Linton SJ, Boersma K: Early identification of patients at risk of developing a persistent back problem: the predictive validity of the Orebro Musculoskeletal Pain Questionnaire. Clin J Pain 2003, 19(2):80-86.
84. Linton SJ, Hallden K: Can we screen for problematic back pain? A screening questionnaire for predicting outcome in acute and subacute back pain. Clin J Pain 1998, 14(3):209-215.
85. Hockings RL, McAuley JH, Maher CG: A systematic review of the predictive ability of the Orebro Musculoskeletal Pain Questionnaire. Spine 2008, 33(15):E494-500.
86. Grotle M, Vollestad NK, Brox JI: Screening for yellow flags in first-time acute low back pain: reliability and validity of a Norwegian version of the Acute Low Back Pain Screening Questionnaire. Clin J Pain 2006, 22(5):458-467.
87. EuroQol-Group: EuroQol–a new facility for the measurement of health-related quality of life. The EuroQol Group. Health Policy 1990, 16(3):199-208.
88. Dolan P: Modeling valuations for EuroQol health states. Med Care 1997, 35(11):1095-1108.
89. Solberg TK, Olsen JA, Ingebrigtsen T, Hofoss D, Nygaard OP: Health-related quality of life assessment by the EuroQol-5D can provide cost-utility data in the field of low-back surgery. Eur Spine J 2005, 14(10):1000-1007.
90. Linde L, Sorensen J, Ostergaard M, Horslev-Petersen K, Hetland ML: Healthrelated quality of life: validity, reliability, and responsiveness of SF-36, 15D, EQ-5D [corrected] RAQoL, and HAQ in patients with rheumatoid arthritis. J Rheumatol 2008, 35(8):1528-1537.
91. Hurst NP, Kind P, Ruta D, Hunter M, Stubbings A: Measuring health-related quality of life in rheumatoid arthritis: validity, responsiveness and reliability of EuroQol (EQ-5D). Br J Rheumatol 1997, 36(5):551-559.
92. van der Roer N, van Tulder M, van Mechelen W, de Vet H: Economic evaluation of an intensive group training protocol compared with usual care physiotherapy in patients with chronic low back pain. Spine 2008, 33(4):445-451.
93. Thiyagarajan A, James TG, Marzo RR. Psychometric properties of the 21-item Depression, Anxiety, and Stress Scale (DASS-21) among Malaysians during COVID-19: a methodological study. Humanit Soc Sci Commun. 2022;9(1):220. doi: 10.1057/s41599-022-01229-x
94. Lee D. The convergent, discriminant, and nomological validity of the Depression Anxiety Stress Scales-21 (DASS-21). J Affect Disord. 2019 Dec 1;259:136-142. doi: 10.1016/j.jad.2019.06.036
95. Nicholas MK. The pain self-efficacy questionnaire: Taking pain into account. Eur J Pain. 2007 Feb;11(2):153-63. doi: 10.1016/j.ejpain.2005.12.008
96. Dubé MO, Langevin P, Roy JS. Measurement properties of the Pain Self-Efficacy Questionnaire in populations with musculoskeletal disorders: a systematic review. Pain Rep. 2021 Dec 21;6(4):e972. doi: 10.1097/PR9.0000000000000972
97. Sullivan MJL, Bishop SR, Pivik J. The Pain Catastrophizing Scale: Development and Validation. Psychol Assess, 1995; 7(4): 524-32.
98. Osman A, Barrios FX, Kopper BA, Hauptmann W, Jones J, O'Neill E. Factor structure, Reliability, and Validity of the Pain Catastrophizing Scale. J Behav Med, 1997; 20(6): 589-605.
99. Bastien CH, Vallières A, Morin CM. Validation of the Insomnia Severity Index as an outcome measure for insomnia research. Sleep Med. 2001 Jul;2(4):297-307. doi: 10.1016/s1389-9457(00)00065-4
100. Yang M, Morin CM, Schaefer K, Wallenstein GV. Interpreting score differences in the Insomnia Severity Index: using health-related outcomes to define the minimally important difference. Curr Med Res Opin. 2009 Oct;25(10):2487-94. doi: 10.1185/03007990903167415
101. Tang NK, Wright KJ, Salkovskis PM. Prevalence and correlates of clinical insomnia co-occurring with chronic back pain. J Sleep Res. 2007 Mar;16(1):85-95. doi: 10.1111/j.1365-2869.2007.00571.x
102. Neblett, R, Hartzell, MM, Cohen, H, Mayer, TG, Williams, M, Choi, YH. Ability of the Central Sensitization Inventory to Identify Central Sensitivity Syndromes in an Outpatient Chronic Pain Sample. The Clinical Journal of Pain 2014; 31(4).
103. Mayer, TG, Neblett, R, Cohen, H, Howard, KJ, Choi, YH, Williams, MJ, Perez, Y, Gatchel, RJ. The Development and Psychometric Validation of the Central Sensitization Inventory (CSI). Pain Pract 2012; 12(4): 276-85.
104. Scerbo T, Colasurdo J, Dunn S, Unger J, Nijs J, Cook C. Measurement Properties of the Central Sensitization Inventory: A Systematic Review. Pain Pract. 2018 Apr;18(4):544-554. doi: 10.1111/papr.12636
105. Cuesta-Vargas, A, Neblett, R, Chiarotto, A, Kregel, J, Nijs, J, Van Wilgen, CP. Dimensionality and Reliability of the Central Sensitization Inventory (CSI) in a Pooled Multi-Country Sample. Journal of Pain 2018; 19(3): 317-29
106. Ford JJ, Kaddour O, Gonzales M, Page P, Hahne AJ. Clinical features as predictors of histologically confirmed inflammation in patients with lumbar disc herniation with associated radiculopathy. BMC Musculoskelet Disord. 2020 Aug 21;21(1):567. doi: 10.1186/s12891-020-03590-x
107. Devilly GJ, Borkovec TD. Psychometric properties of the credibility/expectancy questionnaire. J Behav Ther Exp Psychiatry. 2000 Jun;31(2):73-86. doi: 10.1016/s0005-7916(00)00012-4
108. Smeets RJ, Beelen S, Goossens ME, Schouten EG, Knottnerus JA, Vlaeyen JW. Treatment expectancy and credibility are associated with the outcome of both physical and cognitive-behavioral treatment in chronic low back pain. Clin J Pain. 2008 May;24(4):305-15. doi: 10.1097/AJP.0b013e318164aa75
109. Bagg MK, Wand BM, Cashin AG, Lee H, Hübscher M, Stanton TR, O'Connell NE, O'Hagan ET, Rizzo RRN, Wewege MA, Rabey M, Goodall S, Saing S, Lo SN, Luomajoki H, Herbert RD, Maher CG, Moseley GL, McAuley JH. Effect of Graded Sensorimotor Retraining on Pain Intensity in Patients With Chronic Low Back Pain: A Randomized Clinical Trial. JAMA. 2022 Aug 2;328(5):430-439. doi: 10.1001/jama.2022.9930
110. Richmond H, Hall AM, Hansen Z, Williamson E, Davies D, Lamb SE. Exploring physiotherapists' experiences of implementing a cognitive behavioural approach for managing low back pain and identifying barriers to long-term implementation. Physiotherapy. 2018 Mar;104(1):107-115. doi: 10.1016/j.physio.2017.03.007
111. Ris I, Boyle E, Myburgh C, Hartvigsen J, Thomassen L, Kongsted A. Factors influencing implementation of the GLA:D Back, an educational/exercise intervention for low back pain: a mixed-methods study. JBI Evid Implement. 2021 May 10;19(4):394-408. doi: 10.1097/XEB.0000000000000284
112. Enthoven P, Eddeborn F, Abbott A, Schröder K, Fors M, Öberg B. Patients' experiences of the BetterBack model of care for low back pain in primary care - a qualitative interview study. Int J Qual Stud Health Well-being. 2021 Dec;16(1):1861719. doi: 10.1080/17482631.2020.1861719
113. Adje M, Steinhäuser J, Stevenson K, Mbada CE, Karstens S. Patients' and physiotherapists' perspectives on implementing a tailored stratified treatment approach for low back pain in Nigeria: a qualitative study. BMJ Open. 2022 Jun 20;12(6):e059736. doi: 10.1136/bmjopen-2021-059736
114. Elkins MR, Moseley AM. Intention-to-treat analysis. J Physiother. 2015 Jul;61(3):165-7. doi: 10.1016/j.jphys.2015.05.013
115. Ramsey S, Willke R, Briggs A, et al. Good research practices for cost-effectiveness analysis alongside clinical trials: the ISPOR RCT-CEA Task Force report. Value Health 2005;8:521–33.
116. Schafer JL, Graham JW. Missing data: our view of the state of the art. Psychol Methods 2002;7:147–77.
117. Spritzler J, DeGruttola VG, Pei L. Two-sample tests of area-under-the-curve in the presence of missing data. Int J Biostat 2008;4: Article 1.
118. Simoens S. Health economic assessment: a methodological primer. Int J Environ Res Public Health 2009;6:2950–66.
119. Nixon RM, Wonderling D, Grieve RD. Non-parametric methods for cost-effectiveness analysis: the central limit theorem and the bootstrap compared. Health Econ 2010;19:316–33.
120. Fenwick E, Marshall DA, Levy AR, et al. Using and interpreting cost-effectiveness acceptability curves: an example using data from a trial of management strategies for atrial fibrillation. BMC Health Serv Res 2006;6:52
121. Foster NE, Mullis R, Hill JC, et al. Effect of stratified care for low back pain in family practice (IMPaCT Back): a prospective population-based sequential comparison. Ann Fam Med. 2014 Mar-Apr;12(2):102-11. doi: 10.1370/afm.1625
122. Bowen, D. J., Kreuter, M., Spring, B., Cofta-Woerpel, L., Linnan, L., Weiner, D,Fernandez, M. (2009). How we design feasibility studies. *American Journal of Preventive Medicine, 36*(5), 452-457. doi:10.1016/j.amepre.2009.02.002
123. ADNI. MRI scanner protocols 2022 [Available from:https://adni.loni.usc.edu/methods/documents/mri-protocols/.

| **Signature of the Principal Investigator**  **Name and Designation of**  **Primary Investigator:** | Dr. Kavitha Raja, PT, PhD  Professor and principal  JSS College of Physiotherapy, MG Road, Mysuru-4 |
| --- | --- |
| **Head of the Department:**  **Signature:** |  |
| **Remarks of Chairman and Principal**  **Signature:**  **Date:** |  |

## APPENDIX 1: PHYSICAL EXAMINATION & OUTCOMES

| **PHYSICAL EXAMINATION** |
| --- |
| The physical examination will involve:   1. Observation of the spine for evidence of postural deformity such as a lateral shift or an increased or decreased lumbar lordosis [1], using protocols with acceptable reliability [2,3]. 2. Measurement of lumbar spine active movements into flexion, extension and lateral-flexion using finger-to-floor measurement methods that have been shown to be reliable [4]. 3. Lower limb neurological examination in a seated position, which will involve testing of knee jerk and ankle jerk reflexes, myotomal strength testing and dermatomal sensation in response to light touch with a tissue [5]. Acceptable reliability for these tests has been demonstrated in people with suspected lower limb nerve root compression [5]. 4. Straight leg raise and crossed straight leg raise, which will be considered positive if the participant’s usual lower limb symptoms are reproduced at any angle during passive raising of either leg by the examiner [5]. The reliability of this test is considered good when performed on people with suspected nerve root compression [5]. 5. Prone knee flexion test, which will be considered positive if the participant’s usual anterior thigh symptoms are reproduced at any angle [6,7]. The reliability of this test has been shown to be good in people with suspected nerve root compression [5]. 6. Lumbar spine palpation performed with the participant prone, with the examiner applying pressure centrally over the lumbar spinous processes and unilaterally over the lumbar zygapophyseal joints and/or transverse processes [8]. Where a localised painful or stiff segment is identified, a “mini-treatment” will be undertaken consisting of a 30-second low grade mobilization of the joint, and the participant’s response will be recorded in terms of any changes in pain or range of motion upon repeat testing [9,8]. Studies have demonstrated good reliability for lumbar spine palpation [10,11]. 7. Mechanical loading strategies including sustained prone positioning and repeated extension movements in a prone position (with or without lateral shift of the pelvis), which will be assessed to determine whether a directional preference is present. Directional preference will be defined as the direction of movements or postures that result in either centralisation of symptoms, sustained decrease in symptoms (by at least 1 point on a 0-10 numerical rating scale) or improvement in range-of-motion following the assessment of mechanical loading strategies [12-14]. Assessing for a directional preference has been shown to be reliable [14,15]. 8. Determination of the participant’s ability to activate the transversus abdominis via a localised inward movement of the lower abdominal wall in a standing position which will be visualised and palpated using a reliable method [16,17]. Each participant’s ability to activate the lumbar multifidus via localised generation of tension will also be assessed via palpation of the participant in prone. Although therapist palpation is one recommended method of assessing the activation of multifidus [17], studies evaluating the reliability of this method could not be located. |
| **References** |
| 1. McKenzie R, May S: The Lumbar Spine: Mechanical Diagnosis and Therapy. Waikanae: Spinal Publications Ltd; 2003. 2. Razmjou H, Kramer JF, Yamada R: Intertester reliability of the McKenzie evaluation in assessing patients with mechanical low-back pain. J Orthop Sports Phys Ther 2000, 30(7):368-383. 3. Kilby J, Stigant M, Roberts A: The reliability of back pain assessment by physiotherapists, using a ‘McKenzie algorithm’. Physiotherapy 1990, 76:579-583. 4. Hahne AJ, Keating JL, Wilson SC: Do within-session changes in pain intensity and range of motion predict between-session changes in patients with low back pain? Aust J Physiother 2004, 50(1):17-23. 5. Vroomen PC, de Krom MC, Knottnerus JA: Consistency of history taking and physical examination in patients with suspected lumbar nerve root involvement. Spine 2000, 25(1):91-96. 6. Herron LD, Pheasant HC: Prone knee-flexion provocative testing for lumbar disc protrusion. Spine 1980, 5(1):65-67. 7. Borenstein DG, Wiesel SW, Boden SD: Low back and neck pain: Comprehensive diagnosis and management. Philadelphia: Saunders; 2004. 8. Maitland GD, Hengeveld E, Banks K, English K: Maitland’s Vertebral Manipulation. Elsevier Butterworth Heinemann, 7 2005. 9. Ford JJ, Thompson SL, Hahne AJ: A classification and treatment protocol for low back disorders: Part 1- Specific manual therapy. In press 2011. 10. van Tulder MW, Koes B, Malmivaara A: Outcome of non-invasive treatment modalities on back pain: an evidence-based review. Eur Spine J 2006, 15(Suppl 1):S64-81. 11. Chou R, Atlas SJ, Stanos SP, Rosenquist RW: Nonsurgical interventional therapies for low back pain: a review of the evidence for an American Pain Society clinical practice guideline. Spine 2009, 34(10):1078-1093. 12. Werneke MW: “Centralization” and “directional preference” are not synonymous. J Orthop Sports Phys Ther 2009, 39(11):827. 13. Werneke MW, Hart DL, Cutrone G, Oliver D, McGill T, Weinberg J, Grigsby D, Oswald W, Ward J: Association Between Directional Preference and Centralization in Patients With Low Back Pain. J Orthop Sports Phys Ther 2011, 41(1):22-31. 14. Wetzel FT, Donelson R: The role of repeated end-range/pain response assessment in the management of symptomatic lumbar discs. Spine J 2003, 3(2):146-154. 15. Fritz JM, Delitto A, Vignovic M, Busse RG: Interrater reliability of judgments of the centralization phenomenon and status change during movement testing in patients with low back pain. Arch Phys Med Rehabil 2000, 81(1):57-61. 16. Costa LO, Costa Lda C, Cancado RL, Oliveira Wde M, Ferreira PH: Short report: intra-tester reliability of two clinical tests of transversus abdominis muscle recruitment. Physiother Res Int 2006, 11(1):48-50. 17. Richardson C, Hodges PW, Hides J: Therapeutic exercise for lumbopelvic stabilisation: A motor control approach for the treatment and prevention of low back pain. Edinburgh: Churchill Livingstone, 2nd 2004. |

## APPENDIX: PATIENT REPORTED OUTCOMES

| **INITIAL PATIENT QUESTIONNAIRE** | | | | | | | | | | | |
| --- | --- | --- | --- | --- | --- | --- | --- | --- | --- | --- | --- |
| **Your details Patient Trial ID number _______________** | | | | | | | | | | | |
| **Title** | Mr | Mrs | | Family name (surname) | | | | | **Given name(s)** | | |
|  | Ms | Miss | |  |  |  |  |  |  |  |  |
| **Gender** | |  | | Date of birth *(dd/mm/yyyy)*__ __ /__ __ / __ __ __ __ | | | | | Today’s date *(dd/mm/yyyy)*__ __ /__ __ / __ __ __ __ | | |
|  | Male | Female | |  |  |  |  |  |  |  |  |
| Level of education | Primary | Secondary | | Tertiary (university) | | | | | None | | |
| What is your occupation? …………………………………………………………………………………………………… | | | | | | | | | | | |
| Annual household income?………………………………………………………………………………………………. | | | | | | | | | | | |
| Home address ………………………….………………………………………………………………………………………………………………………………. ………………………………………………………………………………………………………………………………………………………………….……………….  **Phone:** Home: ……………………………… Work: ……………………………….……….… Mobile: …………………………………..………………  **Email** ……………………………………………………………………………………………………………………………………………………………………….. | | | | | | | | | | | |
| **Country of Birth…………………………………………………………………………………………………………………….** | | | | | | | | | | | |
| **Do you require an interpreter?** | | | | | Yes | No | | | | | |
| *If you answered yes, please specify the language*………………………………………………………………… | | | | | | | | | | | |
| **Are you hearing or sight impaired?** | | | | | Yes | No | | | | | |
| **Do you require help with written or spoken communication?** | | | | | | | Yes | | | No | |
| **Tobacco use status (including smoking):**  Current user | | | | | | | Previous user | | | No | |
| **Height** *(in cm)* | | | **Weight** *(in kg)* | | | | | | | | |
| **Is there a compensation case relating to this episode?** | | | | | | | Yes | | | No | |
| **Marital status** | | | | Married | | | Unmarried | | | | |
|  |  |  |  | Divorced | | | Widow / widower | | | | |
| **How did your main pain begin?** | | | | | | | | | | | |
| Injury at home | | | | Car crash | | |  | | | | |
| Injury at work/school | | | | No obvious cause | | | |  | | | |
| Injury in another setting | | | |  | | |  | | | | |
| **How long has your main pain been present?** *(Tick* ***one*** *box only)* | | | | | | | | | | | |
| Less than 3 months | | | | 12 months to 2 years | | | More than 5 years | | | | |
| 3 to 12 months | | | | 2 to 5 years | | |  | | | | |
| Which statement best describes your pain? *(Tick* ***one*** *box only)* | | | | | | | | | | | |
| Always present (always the same intensity) | | | | | | | | | | | |
| Always present (level of pain varies) | | | | | | | | | | | |
| Often present (pain free periods last less than 6 hours) | | | | | | | | | | | |
| Occasionally present (pain occurs once to several times per day, lasting up to an hour) | | | | | | | | | | | |
| Rarely present (pain occurs every few days or weeks) | | | | | | | | | | | |
| **Do you have any of the following?** | | | | | | | | | | | |
| A mental health condition, in particular:  PTSD Anxiety   Depression  Other *(please specify)*………………………………………………………………………………………………… | | | | | | | | | | | |
| Arthritis *(including Rheumatoid/Osteoarthritis)* | | | | | | | | | | | |
| Muscle, bone and joint problems other than arthritis *(including Osteoporosis, Fibromyalgia)* | | | | | | | | | | | |
| Heart and circulation problems *(including Heart Disease, Pacemaker, Blood Disease)*  In particular specify if you have:  High Blood Pressure  High Cholesterol | | | | | | | | | | | |
| Diabetes | | | | | | | | | | | |
| Digestive problems *(including IBS, GORD, Stomach Ulcers, Reflux, Bowel Disease)* | | | | | | | | | | | |
| Respiratory problems *(including Asthma, Lung Disease, COPD, Sleep Apnoea)* | | | | | | | | | | | |
| Neurological problems *(including Stroke, Epilepsy, Multiple Sclerosis, Parkinson’s Disease)* | | | | | | | | | | | |
| Cancer | | | | | | | | | | | |
| Liver, kidney and pancreas problems *(including Pancreatitis, Kidney Disease)* | | | | | | | | | | | |
| Thyroid problems *(including Hyperactive or Hypoactive Thyroid, Graves’ Disease)* | | | | | | | | | | | |
| Any other medical conditions *(please specify)*………………………………………………………………… | | | | | | | | | | | |
| **Health care *(other than your visits to this clinic)*** | | | | | | | | | | | |
| 1. How many times in the past 3 months have you seen a general practitioner about your pain? | | | | | | | | | | | ……… times |
| 1. How many times in the past 3 months have you seen a medical specialist (e.g. orthopaedic surgeon) in regard to your pain? | | | | | | | | | | | ……… times |
| 1. How many times in the past 3 months have you seen health professionals other than doctors (e.g. physiotherapist, chiropractor, psychologist) in regard to your pain? | | | | | | | | | | | ……… times |
| 1. How many times in the past 3 months have you visited a hospital emergency department in regard to your pain? *(Include all visits, regardless of whether or not you were admitted to the hospital from the emergency department)* | | | | | | | | | | | ……… times |
| 1. How many times in the past 3 months have you been admitted to hospital because of your pain? | | | | | | | | | | | ……… times |
| 1. How many diagnostic tests (e.g. X-rays, scans) have you had in the last   3 months relating to your pain? …………. tests   1. Have you ever had surgery on your back?  Yes  No 2. Have you ever had injections into your back?  Yes  No | | | | | | | | | | |  |

| Prior to commencing in this trial, how satisfied have you been with your overall healthcare / treatment for your current episode of low back pain or leg symptoms (sciatica)? (tick one box)   Very  Somewhat  Neither satisfied  Somewhat  Very  dissatisfied dissatisfied nor dissatisfied satisfied satisfied |
| --- |

Please rate your **back** pain by circling the number below that best describes your pain on average over the last week

0 1 2 3 4 5 6 7 8 9 10

*No pain Worst pain possible*

4. Please rate your **leg** pain by circling the number below that best describes your pain on average over the last week

0 1 2 3 4 5 6 7 8 9 10

*No pain Worst pain possible*

| Your work | | | |
| --- | --- | --- | --- |
| **Are you currently employed (have a job to go back to with an employer)?** | | | |
| Yes - If *yes*, are your normal hours:  Working full-time  Working part-time  Please answer the questions below | | No - If *no*, are you:  (tick ***one*** only, then go straight to **Section 3**)  Unable to work due to a condition other than pain  Unable to work due to pain  Not working by choice (student, retired, homemaker)  Seeking employment (I consider myself able to work but cannot find a job) | |
| **During the past seven days, how many hours did you miss from your normal work hours because of problems associated with your pain?**  (Include hours you missed on sick days, times you went in late, left early, etc. because of your pain. *Do not include time you missed to attend this pain clinic).* | | | …………… hours |
| **During the past seven days, how many hours did you actually work?** *(If ‘0’ skip the next question and go to* ***Section 3****)* | | | …………… hours |
| **During the past seven days, how much did your pain affect your productivity while you were working?**  Think about days you were limited in the amount or kind of work you could do, days you accomplished less than you would like, or days you could not do your work as carefully as usual.  If pain affected your work only a little, choose a low number.  Choose a high number if pain affected your work a great deal.  Consider only how much pain affected  productivity while you were working | | | |
| Pain had no effect on my work | 0 1 2 3 4 5 6 7 8 9 10  CIRCLE A NUMBER | | Pain completely prevented me  from working |

| Medication use | | | |
| --- | --- | --- | --- |
| Are you taking any medications?  No *(please go to* ***Section 4****)*  Yes *(Please list all the medications you are taking. Include both prescription and over-the-counter medicines)* | | | |
| Medicine name  *(as on the label)* | Medicine strength *(as on the label)* | How many do you take per day? | How many days per week do you take this medication? |
|  |  |  |  |
|  |  |  |  |
|  |  |  |  |
|  |  |  |  |
|  |  |  |  |
|  |  |  |  |
|  |  |  |  |
|  |  |  |  |
|  |  |  |  |
|  |  |  |  |
|  |  |  |  |
|  |  |  |  |
|  |  |  |  |
|  |  |  |  |
|  |  |  |  |
|  |  |  |  |

| Brief Pain Inventory - Pain intensity and interference | | | | | | | | | | | | | | | | | | | | | | | | | | | | | | | | |  |
| --- | --- | --- | --- | --- | --- | --- | --- | --- | --- | --- | --- | --- | --- | --- | --- | --- | --- | --- | --- | --- | --- | --- | --- | --- | --- | --- | --- | --- | --- | --- | --- | --- | --- |
| **On the diagram below, shade in ALL the areas where you feel pain.** 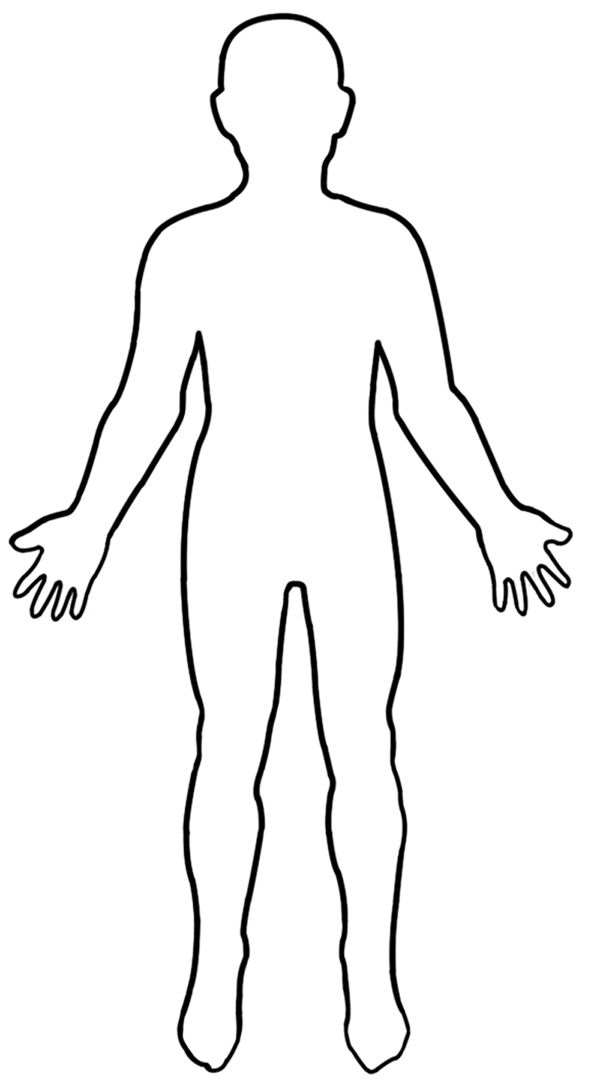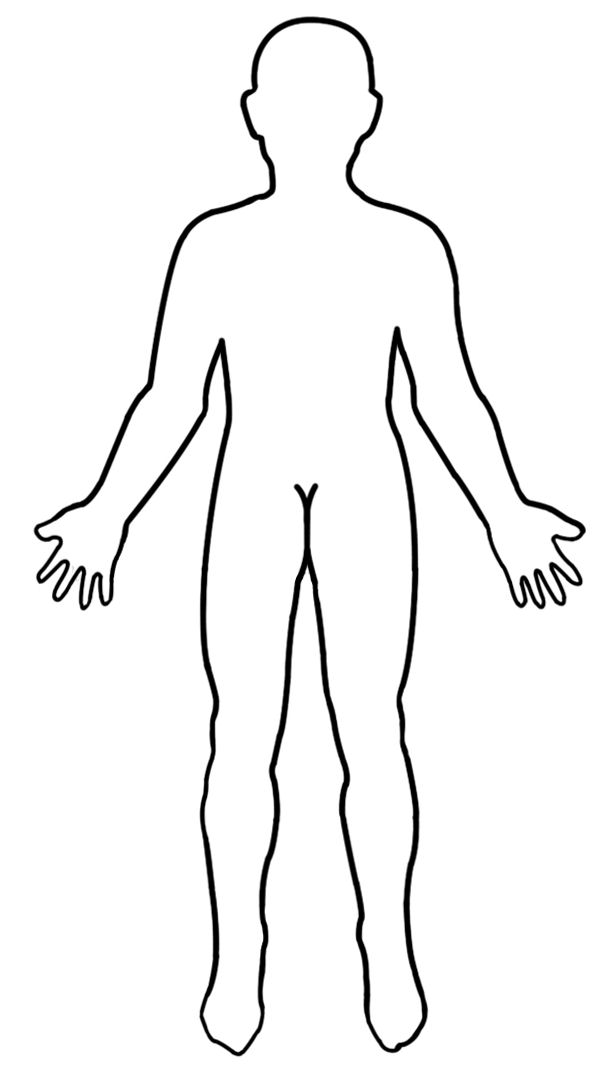 FRONT VIEW  BACK VIEW  LEFT  RIGHT  RIGHT | | | | | | | | | | | | | | | | | | | | | | | | | | | | | | | | |  |
| **On the diagram below, put an X on the ONE area that hurts most.** 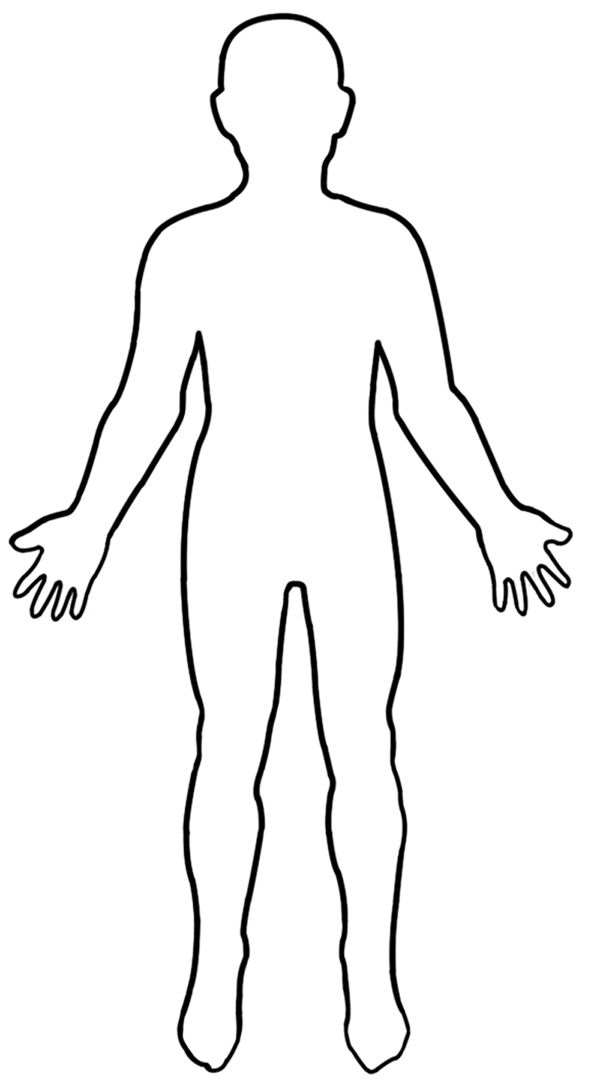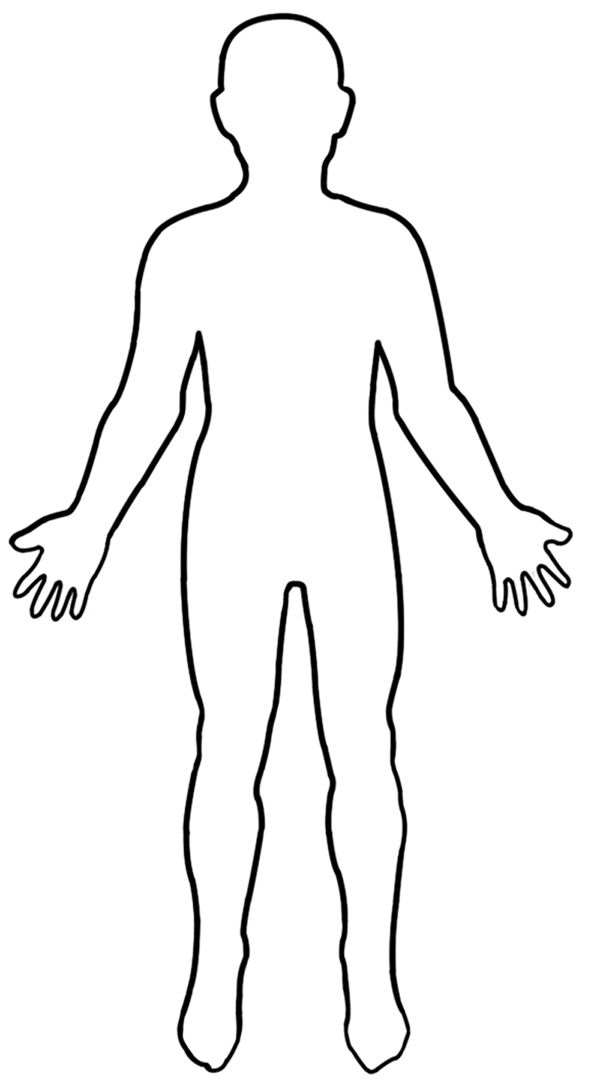 FRONT VIEW  BACK VIEW  LEFT  RIGHT  RIGHT | | | | | | | | | | | | | | | | | | | | | | | | | | | | | | | | |  |
| **Please rate your pain by circling the one number that best describes the following:** | | | | | | | | | | | | | | | | | | | | | | | | | | | | | | | | | |
| 1. Your overall pain at its *worst* in the last week? | 0 | 1 | | 2 | | | | 3 | | 4 | | | 5 | | | | 6 | | | | 7 | | 8 | | | | 9 | | | | 10 | | |
|  | No pain | |  | |  | | | | | | |  | | |  | | | |  | | | | | | Pain as bad as you can imagine | | | | | | | | |
| 1. Your overall pain at its *least* in the last week? | 0 | 1 | | 2 | | | | | 3 | | 4 | | | 5 | | | | 6 | | | | 7 | | 8 | | | | 9 | | | | 10 | |
|  | No pain | |  | |  | | | | | | |  | | |  | | | |  | | | | | | Pain as bad as you can imagine | | | | | | | | |
| 1. Your pain on *average* in the last week? | 0 | 1 | | 2 | | | | 3 | | 4 | | | 5 | | | | 6 | | | | 7 | | 8 | | | | 9 | | | 10 | | | |
|  | No pain | |  | | |  | | | | | |  | | | |  | | | |  | | | | | Pain as bad as you can imagine | | | | | | | | |
| 1. How much overall pain do you have *right now*? | 0 | 1 | | 2 | | | | 3 | | 4 | | | 5 | | | | 6 | | | | 7 | | 8 | | | 9 | | | 10 | | | | |
|  | No pain | |  | | | |  | | | | |  | | | |  | | | |  | | | | | Pain as bad as you can imagine | | | | | | | | |

| **During the past week, how much has pain interfered with the following:** | | | | | | | | | | | | | | | | |
| --- | --- | --- | --- | --- | --- | --- | --- | --- | --- | --- | --- | --- | --- | --- | --- | --- |
| 1. Your general activity? | 0 | 1 | | 2 | 3 | 4 | | 5 | | 6 | | 7 | 8 | | 9 | 10 |
|  | Does not interfere | |  | | | |  | |  | |  | | | Completely interferes | | |
| 1. Your mood? | 0 | 1 | | 2 | 3 | 4 | | 5 | | 6 | | 7 | 8 | | 9 | 10 |
|  | Does not  interfere | |  | | | |  | |  | |  | | | Completely interferes | | |
| 1. Your walking ability? | 0 | 1 | | 2 | 3 | 4 | | 5 | | 6 | | 7 | 8 | | 9 | 10 |
|  | Does not  interfere | |  | | | |  | |  | |  | | | Completely interferes | | |
| 1. Your normal work (both outside the home and housework)? | 0 | 1 | | 2 | 3 | 4 | | 5 | | 6 | | 7 | 8 | | 9 | 10 |
|  | Does not  interfere | |  | | | |  | |  | |  | | | Completely interferes | | |
| 1. Your relations with other people? | 0 | 1 | | 2 | 3 | 4 | | 5 | | 6 | | 7 | 8 | | 9 | 10 |
|  | Does not  interfere | |  | | | |  | |  | |  | | | Completely interferes | | |
| 1. Your sleep? | 0 | 1 | | 2 | 3 | 4 | | 5 | | 6 | | 7 | 8 | | 9 | 10 |
|  | Does not  interfere | |  | | | |  | |  | |  | | | Completely interferes | | |
| 1. Your enjoyment of life? | 0 | 1 | | 2 | 3 | 4 | | 5 | | 6 | | 7 | 8 | | 9 | 10 |
|  | Does not  interfere | |  | | | |  | |  | |  | | | Completely interferes | | |

**OSWESTRY DISABILITY QUESTIONNAIRE**

This questionnaire has been designed to give us information as to how your back or leg pain is affecting your ability to manage in everyday life. Please answer by checking **one box in each section** for the statement which best applies to you. We realise you may consider that two or more statements in any one section apply but please just shade out the spot that indicates the statement ***which most clearly describes your problem.***

**Section 1: Pain Intensity**

I have no pain at the moment

The pain is very mild at the moment

The pain is moderate at the moment

The pain is fairly severe at the moment

The pain is very severe at the moment

The pain is the worst imaginable at the moment

**Section 2: Personal Care (Washing, Dressing, etc.)**

I can look after myself normally without causing extra pain

I can look after myself normally but it causes extra pain

It is painful to look after myself and I am slow and careful

I need some help but can manage most of my personal care

I need help every day in most aspects of self care

I do not get dressed, wash with difficulty and stay in bed

**Section 3: Lifting**

I can lift heavy weights without extra pain

I can lift heavy weights but it gives me extra pain

Pain prevents me lifting heavy weights off the floor but I can manage if they are conveniently placed e.g. on a table

Pain prevents me lifting heavy weights but I can manage light to medium weights if they are conveniently positioned

I can only lift very light weights

I cannot lift or carry anything

**Section 4: Walking**

Pain does not prevent me walking any distance

Pain prevents me from walking more than 2 kilometres

Pain prevents me from walking more than 1 kilometre

Pain prevents me from walking more than 500 metres

I can only walk using a stick or crutches

I am in bed most of the time

**Section 5: Sitting**

I can sit in any chair as long as I like

I can only sit in my favourite chair as long as I like

Pain prevents me sitting more than one hour

Pain prevents me from sitting more than 30 minutes

Pain prevents me from sitting more than 10 minutes

Pain prevents me from sitting at all

**Section 6: Standing**

I can stand as long as I want without extra pain

I can stand as long as I want but it gives me extra pain

Pain prevents me from standing for more than 1 hour

Pain prevents me from standing for more than 30 minutes

Pain prevents me from standing for more than 10 minutes

Pain prevents me from standing at all

**Section 7: Sleeping**

My sleep is never disturbed by pain

My sleep is occasionally disturbed by pain

Because of pain I have less than 6 hours sleep

Because of pain I have less than 4 hours sleep

Because of pain I have less than 2 hours sleep

Pain prevents me from sleeping at all

**Section 8: Social Life**

My social life is normal and gives me no extra pain

My social life is normal but increases the degree of pain

Pain has no significant effect on my social life apart from limiting my more energetic interests e.g. sport

Pain has restricted my social life and I do not go out as often

Pain has restricted my social life to my home

I have no social life because of pain

**Section 9: Traveling**

I can travel anywhere without pain

I can travel anywhere but it gives me extra pain

Pain is bad but I manage journeys over two hours

Pain restricts me to journeys of less than one hour

Pain restricts me to short necessary journeys under 30 minutes

Pain prevents me from traveling except to receive treatment

**Section 10: Employment/Homemaking**

My normal homemaking/job activities do not cause pain.

My normal homemaking/job activities increase my pain, but I can still perform all that is required of me.

I can perform most of my homemaking/job activities, but pain prevents me from performing more physically stressful activities (eg lifting, vacuuming).

Pain prevents me from doing anything but light duties.

Pain prevents me from doing even light duties.

Pain prevents me from performing any job or homemaking chores

| **Health Questionnaire (EQ-5D-5L)** | |
| --- | --- |
| **Mobility**   - I have no problems in walking about - I have slight problems in walking about - I have moderate problems in walking about - I have severe problems in walking about - I am unable to walk about   **Self-care**   - I have no problems washing or dressing myself - I have slight problems washing or dressing myself - I have moderate problems washing or dressing myself - I have severe problems washing or dressing myself - I am unable to wash or dress myself   **Usual activities** (work, study, housework, family or leisure activity)   - I have no problems doing my usual activities - I have slight problems doing my usual activities - I have moderate problems doing my usual activities - I have severe problems doing my usual activities - I am unable to do my usual activities   **Pain / discomfort**   - I have no pain or discomfort - I have slight pain or discomfort - I have moderate pain or discomfort - I have severe pain or discomfort - I have extreme pain or discomfort   **Anxiety / depression**   - I am not anxious or depressed - I am slightly anxious or depressed - I am moderately anxious or depressed - I am severely anxious or depressed - I am extremely anxious or depressed   **NOTE:** To help people say how good or bad a health state is, we have drawn a scale (rather like a thermometer) on which the best state you can imagine is marked 100 and the worst state you can imagine is marked 0. | We would like you to indicate on this scale how good or bad your own health is today, in your opinion. Please do this by drawing a line from the box below to whichever point on the scale indicates how good or bad your health state is today.  9 0  8 0  7 0  6 0  5 0  4 0  3 0  2 0  1 0  100  Worst  imaginable  health state  0  Best  imaginable  health state  Your own  health state  today |

| Depression Anxiety and Stress Scale-21 | | | | |
| --- | --- | --- | --- | --- |
| Please read each statement and circle a number 0, 1, 2 or 3 which indicates how much the statement applied to you *over the past week*. There are no right or wrong answers. Do not spend too much time on any statement.  *The rating scale is as follows:*  0 Did not apply to me at all  1 Applied to me to some degree, or some of the time  2 Applied to me to a considerable degree, or a good part of the time  3 Applied to me very much, or most of the time | | | | |
|  | Not at all | Some of the time | A good part of the time | Most of the time |
| 1. I found it hard to wind down | 0 | 1 | 2 | 3 |
| 1. I was aware of dryness of my mouth | 0 | 1 | 2 | 3 |
| 1. I couldn’t seem to experience any positive feeling at all | 0 | 1 | 2 | 3 |
| 1. I experienced breathing difficulty (e.g. excessively rapid breathing, breathlessness in the absence of physical exertion) | 0 | 1 | 2 | 3 |
| 1. I found it difficult to work up the initiative to do things | 0 | 1 | 2 | 3 |
| 1. I tended to overreact to situations | 0 | 1 | 2 | 3 |
| 1. I experienced trembling (e.g. in the hands) | 0 | 1 | 2 | 3 |
| 1. I felt that I was using a lot of nervous energy | 0 | 1 | 2 | 3 |
| 1. I was worried about situations in which I might panic and make a fool of myself | 0 | 1 | 2 | 3 |
| 1. I felt that I had nothing to look forward to | 0 | 1 | 2 | 3 |
| 1. I found myself getting agitated | 0 | 1 | 2 | 3 |
| 1. I found it difficult to relax | 0 | 1 | 2 | 3 |
| 1. I felt down-hearted and blue | 0 | 1 | 2 | 3 |
| 1. I was intolerant of anything that kept me from getting on with what I was doing | 0 | 1 | 2 | 3 |
| 1. I felt I was close to panic | 0 | 1 | 2 | 3 |
| 1. I was unable to become enthusiastic about anything | 0 | 1 | 2 | 3 |
| 1. I felt I wasn’t worth much as a person | 0 | 1 | 2 | 3 |
| 1. I felt that I was rather touchy | 0 | 1 | 2 | 3 |
| 1. I was aware of the action of my heart in the absence of physical exertion (e.g. a sense of heart rate increase, heart missing a beat) | 0 | 1 | 2 | 3 |
| 1. I felt scared without any good reason | 0 | 1 | 2 | 3 |
| 1. I felt that life was meaningless | 0 | 1 | 2 | 3 |

| Pain Self-Efficacy Questionnaire | |
| --- | --- |
| Rate how confident you are that you can do the following things **at present** despite the pain. Circle one of the numbers on the scale under each item, where 0 = *Not at all confident* and 6 = *Completely confident*.  Remember this questionnaire is not asking whether or not you have been doing these things, but rather how confident you are that you can do them at present, **despite the pain.** | |
| 1. I can enjoy things, despite the pain | 0 1 2 3 4 5 6 Not at all Completely  confident confident |
| 1. I can do most of the household chores (e.g. tidying up, washing dishes, etc.) despite the pain | 0 1 2 3 4 5 6 Not at all Completely  confident confident |
| 1. I can socialise with my friends or family members as often as I used to do, despite the pain | 0 1 2 3 4 5 6 Not at all Completely  confident confident |
| 1. I can cope with my pain in most situations | 0 1 2 3 4 5 6 Not at all Completely  confident confident |
| 1. I can do some form of work, despite the pain (“work” includes housework, paid and unpaid work) | 0 1 2 3 4 5 6 Not at all Completely  confident confident |
| 1. I can still do many of the things I enjoy doing, such as hobbies or leisure activity, despite the pain | 0 1 2 3 4 5 6 Not at all Completely  confident confident |
| 1. I can cope with my pain without medication | 0 1 2 3 4 5 6 Not at all Completely  confident confident |
| 1. I can still accomplish most of my goals in life, despite the pain | 0 1 2 3 4 5 6 Not at all Completely  confident confident |
| 1. I can live a normal lifestyle, despite the pain | 0 1 2 3 4 5 6 Not at all Completely  confident confident |
| 1. I can gradually become more active, despite the pain | 0 1 2 3 4 5 6 Not at all Completely  confident confident |

| Pain Catastrophising Scale | | | | | |
| --- | --- | --- | --- | --- | --- |
| Everyone experiences painful situations at some point in their lives. Such experiences may include headaches, tooth pain, joint or muscle pain. People are often exposed to situations that may cause pain such as illness, injury, dental procedures or surgery.  We are interested in the types of thoughts and feelings that you have when you are in pain. Listed below are thirteen statements describing different thoughts and feelings that may be associated with pain. Using the scale, please indicate the degree to which you have these thoughts and feelings when you are experiencing pain. | | | | | |
|  | Not at all | To a slight degree | To a moderate degree | To a great degree | All the time |
| 1. I worry all the time about whether the pain will end | 0 | 1 | 2 | 3 | 4 |
| 1. I feel I can’t go on | 0 | 1 | 2 | 3 | 4 |
| 1. It’s terrible and I think it’s never going to get any better | 0 | 1 | 2 | 3 | 4 |
| 1. It’s awful and I feel it overwhelms me | 0 | 1 | 2 | 3 | 4 |
| 1. I feel I can’t stand it anymore | 0 | 1 | 2 | 3 | 4 |
| 1. I become afraid that the pain will get worse | 0 | 1 | 2 | 3 | 4 |
| 1. I keep thinking of other painful events | 0 | 1 | 2 | 3 | 4 |
| 1. I anxiously want the pain to go away | 0 | 1 | 2 | 3 | 4 |
| 1. I can’t seem to keep it out of my mind | 0 | 1 | 2 | 3 | 4 |
| 1. I keep thinking about how much it hurts | 0 | 1 | 2 | 3 | 4 |
| 1. I keep thinking about how badly I want the pain to stop | 0 | 1 | 2 | 3 | 4 |
| 1. There’s nothing I can do to reduce the intensity of the pain | 0 | 1 | 2 | 3 | 4 |
| 1. I wonder whether something serious may happen | 0 | 1 | 2 | 3 | 4 |

# Orebro Musculoskeletal Pain Screening Questionnaire

1. **How long have you had your current pain problem? Tick one**

0-1weeks [1] 1-2 weeks [2] 3-4 weeks [3] 4-5 weeks [4] 6-8 weeks [5]

9-11 weeks [6] 3-6 months [7] 6-9 months [8] 9-12 months [9] over 1 year [10]

1. **How would you rate the pain that you have had during the past week? Circle one.**

0 1 2 3 4 5 6 7 8 9 10

*No pain* *Pain as bad as it could be*

1. **How tense or anxious have you felt in the past week? Circle one. How much have you been bothered by feeling depressed in the past week? Circle one.**

0 1 2 3 4 5 6 7 8 9 10

*Absolutely calm and relaxed* *As tense and anxious as I’ve ever felt*

**4***.* **How much have you been bothered by feeling depressed in the past week? Circle one.**

0 1 2 3 4 5 6 7 8 9 10

*Not at all*  *Extremely*

1. **In your view, how large is the risk that your current pain may become persistent? Circle one.**

0 1 2 3 4 5 6 7 8 9 10

###### *No risk* *Very large risk*

1. **In your estimation, what are the chances that you will be working your normal duties in 3 months? Circle one (10-)**

0 1 2 3 4 5 6 7 8 9 10

###### *No chance* *Very large chance*

Here are some of the things which other people have told us about their pain. For each statement please circle one number from 0 to 10 to say how much physical activities, such as bending, lifting, walking or driving would affect your pain.

1. **An increase in pain is an indication that I should stop what I’m doing until the pain decreases.**

0 1 2 3 4 5 6 7 8 9 10

*Completely disagree* *Completely agree*

1. **I should not do my normal work with my present pain.**

0 1 2 3 4 5 6 7 8 9 10

*Completely disagree* *Completely agree*

Here is a list of 5 activities. Please circle the one number which best describes your current ability to participate in each of these activities (10-)

1. **I can do light work for an hour.**

0 1 2 3 4 5 6 7 8 9 10

*Can’t do it because Can do it without*

*pain problem pain being a problem*

1. **I can sleep at night.**

0 1 2 3 4 5 6 7 8 9 10 *Can’t do it because Can do it without pain problem pain being a problem*

**Insomnia Severity Index (ISI)**

For each question, please CIRCLE/TICK the number that best describes your answer.

*Please rate the CURRENT (ie last 2 weeks) SEVERITY of your insomnia problem(s).*

| **Insomnia Problem** | None | Mild | Moderate | Severe | Very severe |
| --- | --- | --- | --- | --- | --- |
| 1. Difficulty falling asleep | 0 | 1 | 2 | 3 | 4 |
| 2. Difficulty staying asleep | 0 | 1 | 2 | 3 | 4 |
| 3. Problems waking up too early | 0 | 1 | 2 | 3 | 4 |

**4. How SATISFIED/DISSATISFIED are you with your CURRENT sleep pattern?**

Very Satisfied Satisfied Moderately satisfied Dissatisfied Very Dissatisfied

0 1 2 3 4

**5. How NOTICEABLE to others do you think your sleep problem is in terms of impairing the quality of your life?**

Not at all noticeable A little Somewhat Much Very much noticeable

0 1 2 3 4

**6. How WORRIED/DISTRESSED are you about your current sleep problem?**

Not at all noticeable A little Somewhat Much Very much noticeable

0 1 2 3 4

**7. To what extent do you consider your sleep problem to INTERFERE with your daily functioning (eg daytime fatigue, mood, ability to function at work/daily chores, concentration, memory, mood etc) CURRENTLY?**

Not at all noticeable A little Somewhat Much Very much noticeable

0 1 2 3 4

**Central Sensitisation Inventory**

**Please circle/tick the best response to the right of each statement**

| 1. I feel tired and unrefreshed when I wake from sleeping. | Never | Rarely | Sometimes | Often | Always |
| --- | --- | --- | --- | --- | --- |
| 2. My muscles feel stiff and achy. | Never | Rarely | Sometimes | Often | Always |
| 3. I feel pain all over my body. | Never | Rarely | Sometimes | Often | Always |
| 4. I have headaches. | Never | Rarely | Sometimes | Often | Always |
| 5. I do not sleep well. | Never | Rarely | Sometimes | Often | Always |
| 6. I have difficulty concentrating. | Never | Rarely | Sometimes | Often | Always |
| 7. Stress makes my physical symptoms get worse. | Never | Rarely | Sometimes | Often | Always |
| 8. I have muscle tension in my neck and shoulders. | Never | Rarely | Sometimes | Often | Always |
| 9. I have difficulty remembering things. | Never | Rarely | Sometimes | Often | Always |

| **CLINICAL INFLAMMATION SCORE** | |
| --- | --- |
| 1. **Constant Symptoms**   Are your symptoms constantly with you 24 hours a day even when you rest or gently walk?   yes   no   1. **Morning pain/stiffness**   In the past 3 days have you had a painful or stiff back in the morning?   yes   no  If so, how long does it take for the pain/stiffness to ease if you don't take medication?   Less than 30 minutes   30-60 minutes   greater than 1 hour   constant pain/stiffness all day   I always take medication   1. **Easing factors**   Is your pain EASED by walking short distances (eg. one block)?   yes   no | 1. **Night symptoms**   **4a) Waking**  In the past 3 days how often have the symptoms woken you while sleeping?   not at all   some nights   most nights   every night  **4b) Reason for waking**  In the past 3 days if the symptoms have woken you, what has been the reason?   not applicable   rolling over   wake due to pain without moving   Other  **4c) Return to sleep**  In the past 3 days if the symptoms have woken you, what have you done so you can go back to sleep?  Please tick one box only (the thing you usually do to get back to sleep)   not applicable   nothing helps, can't fall back to sleep   change positions in bed   sit up   get out of bed   take medication   Other |

**Treatment Credibility Questionnaire**

We would like you to indicate below how much you believe, *right now*, that the treatment you are about to receive / are receiving will help you with your back problem.

**1) At this point, how logical does the treatment offered to you seem?**

0 1 2 3 4 5 6 7 8 9 10

*Not at all logical Somewhat logical Very logical*

**2) At this point, how successful do you think this treatment will be in helping you with your back problem?**

0 1 2 3 4 5 6 7 8 9 10

*Not all successful Somewhat successful Very successful*

**3) How confident would you be in recommending this treatment to a friend who experiences similar problems?**

0 1 2 3 4 5 6 7 8 9 10

*Not at all confidence Somewhat confident Very confident*

**4) By the end of your treatment, how much improvement in your back condition do you think will occur?**

0% 10% 20% 30% 40% 50% 60% 70% 80% 90% 100%

*No improvement Moderate improvement Complete improvement*

## APPENDIX 2: CLASSIFICATION INTO SUBGROUPS AND TREATMENT

| **Classification into subgroups ^[1-10]^** |
| --- |
| 1. **Disc herniation with associated radiculopathy** |
| To be classified in this subgroup, participants will have at least 2 of the following 3 diagnostic criteria:   1. referred leg symptoms (below the knee for L4/5 or L5/S1 herniations, or into the anterior thigh for L1/2, L2/3 or L3/4 herniations), 2. reproduction of usual leg symptoms on straight leg raise (SLR) or reproduction of usual anterior thigh symptoms on prone-knee-flexion testing in combination with the Hancock rule [1] comprising at least 3 positives out of 4 clinical examination signs suggestive of radiculopathy: 3. pain location in concordance with a nerve root, 4. corresponding sensory deficit (reduced dermatomal sensation, 5. corresponding reflex deficit (reduced reflex), 6. motor weakness (reduced myotomal strength) [2] and, 7. a Computerised Tomography (CT) or Magnetic Resonance Imaging (MRI) scan demonstrating a lumbar disc herniation consistent with #i or #ii above [3]. |
| 1. **Reducible discogenic pain** |
| To be classified in this subgroup, participants will present with at least 4 out of 9 features indicative of discogenic low back pain. The features have been identified through literature searching, consideration of causal mechanisms and the results of a Delphi study of experts in the field [4]. The features are:  1) Presence of low back pain with or without leg pain  2) Sitting limited to less than 60 minutes  3) Symptoms worse the next morning or next day following the initial injury  4) History of working in a job involving manual handling  5) A mechanism of injury associated with flexion/rotation and/or compression loading  6) At least some difficulty with forward bending  7) At least some difficulty with lifting  8) At least some difficulty with sit-to-stand  9) At least some difficulty with coughing/sneezing.  Participants will also demonstrate a directional preference in response to mechanical loading strategies on physical examination. The presence of a directional preference has been proposed as identifying people likely to have discogenic pain where a posterior or posterolaterally migrated nucleus pulposus can be “reduced” into a more central and non-pain provoking position [5-7]. |
| 1. **Non-reducible discogenic pain** |
| To be classified in this subgroup, participants will have at least 4 out of 9 signs of discogenic pain as outlined above for the reducible discogenic pain subgroup. However, participants who do not demonstrate a directional preference in response to mechanical loading strategies will be classified in the non-reducible (rather than reducible) discogenic pain subgroup provided they do not satisfy the selection criteria for the zygapophyseal joint dysfunction group [8]. |
| 1. **Zygapophyseal joint dysfunction** |
| This group will comprise participants believed to have zygapophyseal joint dysfunction as the primary source of their back symptoms. To be classified in this subgroup, participants will have at least 3 of the following 4 features of zygapophyseal dysfunction:   1. presence of unilateral low back pain, 2. a regular compression pattern (pain reproduced with lumbar extension and ipsilateral lateral-flexion movements), 3. localized pain on ipsilateral passive postero-anterior accessory movement applied through the transverse process or the zygapophyseal joint at one or two segments and, 4. improvement in pain or range-of-movement following a “mini-treatment” of manual therapy directed at the zygapophyseal joint [9]. |
| 1. **No identifiable pathoanatomical** |
| Participants without a clear pathoanatomical classification (ie. They do not fit one of the other four pathoanatomical subgroups), who also have an Orebro Musculoskeletal Pain Questionnaire score greater than 105, will be classified as belonging to this subgroup. In these participants it is hypothesized that psychosocial and/or neurophysiological factors may be negatively impacting their recovery [10]. |
| 1. **Others:**   Participants other pathoanatomical disorder (ie. They do not fit one of the other four pathoanatomical subgroups) |

Additionally, patients in all STOPS subgroups will be further evaluated according to

1. To their dominant pain type (nociplastic, neuropathic or nociceptive) using validated assessment criteria ^[11-16]^.
2. Psychosocial barriers to recovery, evaluated via the Orebro Musculoskeletal Pain Questionnaire^[17]^, Depression, Anxiety and Stress Scale^[18]^, Pain Self-Efficacy Questionnaire^[19]^ and Pain Catastrophising Scale ^[20]^.
3. Suspected presence of inflammation based on a validated symptom screening tool ^[21]^

**References:**

1. Hancock MJ, Koes B, Ostelo R, Peul W. Diagnostic accuracy of the clinical examination in identifying the level of herniation in patients with sciatica. Spine (Phila Pa 1976). 2011;36(11):E712–9.
2. Petersen, T., Laslett, M. & Juhl, C. Clinical classification in low back pain: best-evidence diagnostic rules based on systematic reviews. *BMC Musculoskelet Disord* 18**,** 188 (2017).
3. Berry JA, Elia C, Saini HS, Miulli DE. A Review of Lumbar Radiculopathy, Diagnosis, and Treatment. Cureus. 2019 Oct 17;11(10):e5934. doi: 10.7759/cureus.5934
4. Chan AY, Ford JJ, McMeeken JM, Wilde VE. Preliminary evidence for the features of non-reducible discogenic low back pain: survey of an international physiotherapy expert panel with the Delphi technique. Physiotherapy. 2013 Sep;99(3):212-20. doi: 10.1016/j.physio.2012.09.007
5. Petersen T, Laslett M, Thorsen H, Manniche C, Ekdahl C, Jacobsen S: Diagnostic classification of non-specific low back pain. A new system integrating pathoanatomic and clinical categories. Physiother Theor Prac 2003, 19:213-237.
6. Wetzel FT, Donelson R: The role of repeated end-range/pain response assessment in the management of symptomatic lumbar discs. Spine J 2003, 3(2):146-154.
7. Vining R, Potocki E, Seidman M, Morgenthal AP. An evidence-based diagnostic classification system for low back pain. *J Can Chiropr Assoc*. 2013;57(3):189-204.
8. Ford JJ, Hahne AJ, Chan AYP, Surkitt LD. A classification and treatment protocol for low back disorders. Part 3: functional restoration for intervertebral disc related disorders. Phys Ther Rev 2012; 17(1): 55-75.
9. Ford JJ, Thompson SL, Hahne AJ. A classification and treatment protocol for low back disorders. Part 1: specific manual therapy. Phys Ther Rev 2011; 16(3): 168-77.
10. Ford JJ, Richards MJ, Hahne AJ. A classification and treatment protocol for low back disorders. Part 4: functional restoration for low back disorders associated with multifactorial persistent pain. Phys Ther Rev 2012; 17(5): 322-34
11. Smart, K. M., C. Blake, A. Staines, M. Thacker and C. Doody (2012). "Mechanisms-based classifications of musculoskeletal pain: part 1 of 3: symptoms and signs of central sensitisation in patients with low back (+/- leg) pain." Manual Therapy 17(4): 336-344.
12. Smart, K. M., C. Blake, A. Staines, M. Thacker and C. Doody (2012). "Mechanisms-based classifications of musculoskeletal pain: part 2 of 3: symptoms and signs of peripheral neuropathic pain in patients with low back (+/- leg) pain." Manual Therapy 17(4): 345-351.
13. Smart, K. M., C. Blake, A. Staines, M. Thacker and C. Doody (2012). "Mechanisms-based classifications of musculoskeletal pain: part 3 of 3: symptoms and signs of nociceptive pain in patients with low back (+/- leg) pain." Manual Therapy 17(4): 352-357.
14. Smart KM, Blake C, Staines A, Doody C. Clinical indicators of 'nociceptive', 'peripheral neuropathic' and 'central' mechanisms of musculoskeletal pain. A Delphi survey of expert clinicians. Man Ther. 2010 Feb;15(1):80-7. doi: 10.1016/j.math.2009.07.005.
15. Kosek E, Clauw D, Nijs J, Baron R, Gilron I, Harris RE, Mico JA, Rice AS, Sterling M. Chronic nociplastic pain affecting the musculoskeletal system: clinical criteria and grading system. Pain. 2021 Nov 1;162(11):2629-34.
16. Nijs J, Lahousse A, Kapreli E, Bilika P, Saraçoğlu İ, Malfliet A, Coppieters I, De Baets L, Leysen L, Roose E, Clark J. Nociplastic pain criteria or recognition of central sensitization? Pain phenotyping in the past, present and future. Journal of clinical medicine. 2021 Jul 21;10(15):3203.
17. Hockings RL, McAuley JH, Maher CG: A systematic review of the predictive ability of the Orebro Musculoskeletal Pain Questionnaire. Spine 2008, 33(15):E494-500.
18. Lee D. The convergent, discriminant, and nomological validity of the Depression Anxiety Stress Scales-21 (DASS-21). J Affect Disord. 2019 Dec 1;259:136-142. doi: 10.1016/j.jad.2019.06.036
19. Dubé MO, Langevin P, Roy JS. Measurement properties of the Pain Self-Efficacy Questionnaire in populations with musculoskeletal disorders: a systematic review. Pain Rep. 2021 Dec 21;6(4):e972. doi: 10.1097/PR9.0000000000000972
20. Osman A, Barrios FX, Kopper BA, Hauptmann W, Jones J, O'Neill E. Factor structure, Reliability, and Validity of the Pain Catastrophizing Scale. J Behav Med, 1997; 20(6): 589-605.
21. Ford JJ, Kaddour O, Gonzales M, Page P, Hahne AJ. Clinical features as predictors of histologically confirmed inflammation in patients with lumbar disc herniation with associated radiculopathy. BMC Musculoskelet Disord. 2020 Aug 21;21(1):567. doi: 10.1186/s12891-020-03590-x

***STOPS Physiotherapy***

The following treatment components will be available for treating physiotherapists to select from guided by assessment findings:

1. ***Advice and education***

Throughout the physiotherapy sessions, participants will be provided with education and advice regarding the nature and management of their condition. This will include explanation of pain mechanisms (ie nociceptive, neuropathic or nociplastic pain) and if relevant explanation of the pathoanatomical diagnosis. Information will also be provided regarding prognosis and the proposed mechanisms of treatment effect ^[1]^.

1. ***Goal setting***

Participants in collaboration with the physiotherapist will develop meaningful goals ^[2]^ that will direct a graded exercise and activity program. At the beginning of the program, participants will be asked to identify short and long-term goals regarding activities (e.g., hobbies or work) or other aspects of their life such as relationships or general health. The participant and physiotherapist will then develop a graded activity and exercise program that will specifically target the achievement of these goals ^[1]^.

1. ***Pacing***

Participants may be taught to pace their activities appropriately to avoid exacerbations of their pain due to over or under-activity. This involves assisting participants to find a baseline tolerance for a particular activity, and then initially limiting the activity below the threshold until tolerance has improved. In patients with a primary nociceptive or neuropathic pain mechanism, effective pacing will be a focus prior to commencing graded activity and functional exercise ^[1]^. For patients with a nociplastic dominant pain type pacing strategies will still be explored but with caution not to reinforce unhelpful beliefs on avoidance of activity.

1. ***Management of inflammation***

Participants with validated clinical signs of inflammation ^[3]^ in conjunction with a primary nociceptive or neuropathic pain mechanism will be encouraged to see their pharmacist or medical practitioner regarding anti-inflammatory medication options. Regular walking, lumbar spine taping, and postural modification will also be implemented in an effort to control inflammatory processes. Management of inflammation will be a focus prior to commencing graded activity and exercise.

1. ***Sleep management***

Participants with disturbed sleep due to low back pain will be provided with sleep strategies, such as promotion of a sleep routine, relaxation prior to bed, and appropriate bedding and positioning. Sleeping posture will be explored for patients with a nociceptive/neuropathic dominant pain type. Medication strategies for the management of sleep may also be discussed with the participant’s treating medical practitioner ^[1]^.

1. ***Motor control training***

In participants with a primary nociceptive or neuropathic pain mechanism, lumbo-pelvic motor control strategies will be assessed. In cases where unhelpful motor control is noted specific muscle activation of transversus abdominis and lumbar multifidus ^[4,5]^ will be undertaken. Where necessary this will incorporate relaxation and retraining of over-active muscles. Motor control training will generally commence in a lying position, progress to standing, and then be integrated into functional activities (such as walking, squatting, lifting and bending). Prior to starting graded activity and functional exercise, the focus will be on developing adequate motor control for walking.

Participants may be invited to alter their posture and movements in a way that is conducive to recovery from their particular injury (e.g., avoiding excessive lumbar flexion in the presence of a disc injury with predominant neuropathic pain pattern) or in a way that relieves their pain (e.g., maintaining straighter posture when sitting if this results in pain relief) ^[6]^. Movements and postures will be identified as either helpful or unhelpful based on response to mini-treatment. To facilitate straight postures, tape may be applied to the lower back by the physiotherapist using a previously reported safe protocol ^[6]^.

1. ***Management of psychosocial barriers to recovery***

High priority psychosocial barriers to recovery determined by physiotherapy assessment and baseline screening will be addressed using a variety of strategies ^[6,7]^ including:

- Cognitive restructuring of unhelpful thoughts or beliefs that are expressed by the participant in the baseline assessment and associated questionnaires ^[8]^.
- Education relating to overcoming fear of movement and activity, by understanding that pain in response to gentle activity (such as short walks or mild bending) in persistent low back pain is not indicative of tissue damage, hence these activities do not need to be completely avoided (but perhaps modified or paced appropriately) ^[9]^.
- Some behavioural strategies will also be implemented, such as positive reinforcement of behaviour that is likely to improve recovery of function (eg. encouragement and praise is given to participants when they increase their exercises appropriately or achieve an activity goal) ^[10]^.

1. ***Specific manual therapy***

In participants where a clinical pattern is identified that is indicative of a likely positive response to manual therapy, specific manual therapy will be provided as described in the STOPS specific manual therapy protocol ^[11]^.

1. ***Directional preference management***

In participants where a directional preference is identified, directional preference management (in accordance with the McKenzie method) will be applied in accordance with our directional preference management protocol ^[12]^.

1. ***Graded exercise***

Participants will be instructed in a graded exercise program aiming to improve functional capacity to facilitate achievement of the identified goals. These exercises will be performed in the clinic under the physiotherapist’s supervision as well as at home. Common exercises that may be suitable for participants (based on experience from our previous trial ^[13]^) include: walking, stationary bike, step-ups, bicep curls, forward raises, squats, lunges and lifting. Starting dosage for weights is generally low (0.5 to 2kg) and then progressed gradually depending on the participants goals (eg. a participant with a goal of being able to lift their 15kg child will progress up to 15kg weights for lifting practice). Progressions will be negotiated with the participant and monitored closely by the physiotherapist to ensure correct exercise technique and to avoid unreasonable symptom exacerbation. Participants with a primary nociceptive or neuropathic pain mechanism will progress exercise in a pain contingent manner, whereas those with a primary nociplastic pain mechanism will progress in a negotiated time contingent manner ^[6,8]^.

1. ***Graded activity***

All participants will work collaboratively with the physiotherapist on a graded activity program to facilitate a progressive increase in activities that are limited due to the participant’s low back condition. Over time the participant will progress to targeting medium and long term activity goals thereby increasing engagement with meaningful activities and improving quality of life ^[8]^.

1. ***Management of pain***

Strategies for the management of daily pain will be recommended to participants. These options will include the self-application of ice or heat, and exercises that may relieve pain (such as gentle stretches). Participants will also be encouraged to follow the advice of their pharmacist and/or medical practitioner to ensure that adequate pain medication is available ^[1]^.

1. ***Relaxation strategies***

Strategies to promote relaxation may include encouraging participants to undertake activities that they find relaxing (e.g., listening to music, walking, hobbies), as well as formal relaxation strategies including mindfulness and progressive muscular relaxation ^[1]^.

1. ***Management of increases in pain***

Participants who report a persisting increase in their usual level of symptoms (eg. from an activity they performed at home) will be provided with strategies to manage their increased symptoms. The physiotherapist will help to identify the cause of the increase in pain to avoid repeat occurrences, posture and taping may need to be re-visited, and exercises may need to be temporarily modified until pain resides. For significant and persistent increases in pain, the physiotherapist will reassess the participant, and refer them to their medical practitioner for further investigation or management if necessary ^[1]^.

1. ***Referral to other healthcare providers***

If physiotherapists notice that a participant is not responding well to treatment or possesses barriers to recovery that might require the input of another healthcare practitioner (such as a psychologist if a patient becomes highly depressed), then a referral to their medical practitioner will be made for consideration of additional or alternative intervention ^[1]^.

**Reference**

[1] Hahne AJ, Ford JJ, Surkitt LD, Richards MC, Chan AY, Thompson SL, et al. Specific treatment of problems of the spine (STOPS): design of a randomised controlled trial comparing specific physiotherapy versus advice for people with subacute low back disorders. BMC Musculoskelet Disord. 2011;12:104.

[2] Sowden M, Hatch A, Gray SE, Coombs J. Can four key psychosocial risk factors for chronic pain and disability (Yellow Flags) be modified by a pain management programme? A pilot study. Physiotherapy, 2006. 92(1): p. 43-49.

[3] Ford JJ, Kaddour O, Gonzales M, Page P, Hahne AJ. Clinical features as predictors of histologically confirmed inflammation in patients with lumbar disc herniation with associated radiculopathy. BMC Musculoskelet Disord. 2020 Aug 21;21(1):567. doi: 10.1186/s12891-020-03590-x

[4] O'Sullivan PB, Phyty GD, Twomey LT, Allison GT. Evaluation of specific stabilizing exercise in the treatment of chronic low back pain with radiologic diagnosis of spondylolysis or spondylolisthesis. Spine (Phila Pa 1976). 1997 Dec 15;22(24):2959-67. doi: 10.1097/00007632-199712150-00020

[5] Hides JA, Jull GA, Richardson CA. Long-term effects of specific stabilizing exercises for first-episode low back pain. Spine (Phila Pa 1976). 2001 Jun 1;26(11):E243-8. doi: 10.1097/00007632-200106010-00004

[6] Ford JJ, Hahne AJ, Chan AYP, et al. A classification and treatment protocol for low back disorders. Part 3: functional restoration for intervertebral disc related disorders. Phys Ther Rev 2012;17:55–75.

[7] Surkitt LD, Ford JJ, Chan AY, Richards MC, Slater SL, Pizzari T, Hahne AJ. Effects of individualised directional preference management versus advice for reducible discogenic pain: A pre-planned secondary analysis of a randomised controlled trial. Man Ther. 2016 Sep;25:69-80. doi: 10.1016/j.math.2016.06.002

[8] Ford JJ, Richards MJ, Hahne AJ. A classification and treatment protocol for low back disorders. Part 4: functional restoration for low back disorders associated with multifactorial persistent pain. Phys Ther Rev 2012;17:322–34.

[9] Butler DS. Explain Pain. 2003, Adelaide: Noigroup Publications.

[10] Lindström I, Ohlund C, Eek C, Wallin L, Peterson LE, Fordyce WE, Nachemson AL. The effect of graded activity on patients with subacute low back pain: a randomized prospective clinical study with an operant-conditioning behavioral approach. Phys Ther. 1992 Apr;72(4):279-90; discussion 291-3. doi: 10.1093/ptj/72.4.279

[11] Ford JJ, Thompson SL, Hahne AJ. A classification and treatment protocol for low back disorders. Part 1—specific manual therapy. Phys Ther Rev 2011;16:168–77.

[12] Ford JJ, Surkitt LD, Hahne AJ. A classification and treatment protocol for low back disorders. Part 2: directional preference management for reducible discogenic pain. Phys Ther Rev 2011;16:423–37.

[13] Ford JJ, Hahne AJ, Surkitt LD, Chan AY, Richards MC, Slater SL, Hinman RS, Pizzari T, Davidson M, Taylor NF. Individualised physiotherapy as an adjunct to guideline-based advice for low back disorders in primary care: a randomised controlled trial. Br J Sports Med. 2016 Feb;50(4):237-45. doi: 10.1136/bjsports-2015-095058

## APPENDIX 3: QUALITATIVE INTERVIEWS

**INTERVIEW GUIDE FOR PATIENTS ABOUT BOTH USUAL CARE AND STOPS PROGRAM**

Aims

1. Explore participants’ thoughts and feelings around **their experiences** throughout the 11-week physiotherapy program for their low back pain
2. Compare and contrast perceptions about the current physiotherapy program relative to previous treatments received (if any), and relative to expectations coming in to treatment.
3. Explore participants’ thoughts and feelings about **changes** that they experienced throughout the 11-week treatment program
4. Explore participants’ expectations on their **plans for the future** for managing their low back pain.

**Brief introduction**

Thank-you for participating in the research study. We ask some participants at the end of their treatment to share their thoughts and experiences about the treatment they just received.

**Section 1: Perceptions of the program**

**What are your thoughts about the physiotherapy program you’ve just completed?**

Prompts:

- Can you expand/tell me more about…?
- **Can you talk about what the experience was like for you completing the physiotherapy treatment program….?**
- Can you elaborate on any particular aspects of the physiotherapy program that come to mind……?

**Can you tell me about some of things that you and your physiotherapist talked about regarding your back pain?**

Prompts:

- And what are your thoughts about that…..
- Is there anything else that you recall talking about in relation to your back pain…
- Can you tell me about anything you learnt about managing your back pain….

**Section 2: Perceptions on the treatment relative to previous treatment or expectations**

**Can you tell me about other treatments you have tried previously for your low back condition?**

Prompts (esp. if previous physiotherapy, but also other treatments)

- **How did that treatment compare to the physiotherapy treatment you just received….?**

**Coming into the physiotherapy treatment program, what were your expectations?**

Prompts

- And how did the program you just completed match up with those expectations…..?
- What were you expecting the physiotherapy would involve….?

**Section 3: Perceptions on changes from the program**

**Can you talk about how you feel now compared to before the treatment program?**

Prompts

- Can you expand/tell me more about…?
- (if they mention being better or worse) – in what ways are you better / worse, what has changed…?
- **Can you tell me about 1 or 2 examples of what has changed…?**

**How do you feel now about managing your low back condition? How does that compare to before treatment?**

Prompts

- Can you expand/tell me more about…

**How do you feel now about doing your usual activities? How does that compare to before treatment?**

Prompts

- Can you expand/tell me more about…?Can you give me an example of how 1 or 2 activities are now compared to before treatment?

**Can you tell me what parts of the treatment program contributed to the changes you described above?**

Prompts:

- Which parts of the physiotherapy program contributed most to the differences you described?
- Can you expand/tell me more about…?

**Section 4: Plans for the future**

**Can you tell me about your plans for managing your low back condition in the future**

Prompts

- Can you expand/tell me more about…?
- **Are there be any other treatments that you are considering…?**

**Section 5: Is there anything else you would like to say that we have not covered in the interview?**

**INTERVIEW GUIDE FOR PHYSIOTHERAPISTS ABOUT THE USUAL CARE PROGRAM**

Aims

1. Explore physiotherapists’ thoughts and feelings around **their experiences** delivering physiotherapy for patients with low back pain in the trial.
2. Explore physiotherapists’ thoughts and feelings about patient **responses** to the physiotherapy treatment
3. Explore **barriers and facilitators** to implementing physiotherapy treatment for people with low back pain.

**Brief introduction**

I would like to ask you about the physiotherapy treatment you have been providing for people with low back pain over the last __ months. I want to particularly consider the recent period where you were treating patients in the trial.

**Section 1: Perceptions of the physiotherapy treatment approach**

**Can you share some general thoughts about the physiotherapy treatment you have been providing for people with low back pain?**

Prompts:

- Can you expand/tell me more about…?
- OK, any other thoughts…?

**Can you tell me about what your approach has been to assessing people with low back pain in the trial?**

Prompts:

- Can you expand/tell me more about…?
- Anything else you can tell me about your approach to assessing patients in the trial…?

**Can you talk about the treatment approaches you have used for people with low back pain in the trial?**

Prompts:

- Can you expand/tell me more about…?
- Anything else you can tell me about your approach to treating patients in the trial…?

**Section 2: Perceptions on patient responses to physiotherapy treatment**

**Can you tell me about how patients with low back pain have been responding to your physiotherapy treatment in the trial?**

Prompts

- Tell me about any changes you have seen in patients, or changes they have told you about….?
- **Can you give me 1-2 examples of what changes have occurred in patients with the treatment?**

**Can you tell me what parts of your treatment contributed to the changes described above?**

Prompts:

- Which components of the physiotherapy treatment program seemed to be leading to the changes described above?
- Can you expand/tell me more about…?
- Can you give me an example…?

**Tell me about any feedback patients have been giving you about the physiotherapy treatment program?**

Prompts:

- What did they like or dislike about it…?
- Can you tell me about any difficulties or challenges patients have had with the treatment…?

**Section 3: Perceptions on barriers and facilitators to implementing physiotherapy treatment**

**Can you talk about what it was like for you delivering physiotherapy treatment for patients with low back pain in the trial?**

Prompts:

- Can you expand/tell me more about…?
- Are there any particular components of the treatment that come to mind when you think about your experiences delivering the physiotherapy treatment…?

**Can you tell me about any factors or circumstances that made it easier for you to implement physiotherapy in the trial?**

Prompts:

- Can you expand/tell me more about…?
- **Tell me about *how* that made it easier for you…?**

**Can you tell me about any barriers or challenges to implementing the physiotherapy in the trial?**

Prompts:

- Can you expand/tell me more about…?
- Expand on telling me about *how* that was a challenge for you…?
- Did you manage to find a way to overcome that barrier…?
  - If so, how did you overcome it…?
  - If not, what might help to overcome that barrier…?

**Section 4: Is there anything else you would like to say that we have not covered in the interview?**

**INTERVIEW GUIDE FOR PHYSIOTHERAPISTS ABOUT THE STOPS PROGRAM**

Aims

1. Explore physiotherapists’ thoughts and feelings around **their experiences** completing training in the STOPS physiotherapy approach for low back pain.
2. Explore physiotherapists’ thoughts and feelings around **their experiences** delivering STOPS physiotherapy for patients with low back pain in the trial.
3. Explore physiotherapists’ thoughts and feelings about patient **responses** to the STOPS physiotherapy treatment
4. Explore **barriers and facilitators** to implementing STOPS physiotherapy treatment for people with low back pain.
5. Compare and contrast physiotherapists’ **perceptions** about the STOPS physiotherapy program relative to other physiotherapy methods used in the past
6. Explore physiotherapists’ expectations on their **plans for the future** for assessing and treating low back pain patients.

**Brief introduction**

I would like to ask you about the STOPS physiotherapy treatment you have been providing for people with low back pain over the last __ months. I want to particularly consider the recent period where you were treating patients with STOPS physiotherapy in the trial.

**Section 1: Perceptions of the STOPS physiotherapy training**

**Can you share your thoughts on the STOPS training program that you completed?**

Prompts:

- Can you expand/tell me more about…?
- What elements of the training program come to mind…?

**Can you talk about how you felt once you finished the training, and it was time to start treating patients using the STOPS approach?**

Prompts:

- Can you expand/tell me more about…?
- **How prepared did you feel to start treating patients with STOPS…?**
- **Do any elements of the STOPS treatment approach come to mind when you reflect on your preparedness to start treating…?**

**Section 2: Perceptions of the STOPS physiotherapy assessment and treatment approach**

**Can you share some general thoughts about the STOPS physiotherapy treatment you have been providing for people with low back pain?**

Prompts:

- Can you expand/tell me more about…?
- OK, any other thoughts…?
- **Tell me how the STOPS approach differs from your usual physiotherapy treatment approach…?**

**How has assessing people with low back pain using STOPS been going?**

Prompts:

- Can you expand/tell me more about…?
- **Tell me how the STOPS assessment approach differs from your usual physiotherapy assessment approach…?**

**Can you talk about the treatment approaches you have used for people with low back pain in the trial using STOPS?**

Prompts:

- Can you expand/tell me more about…?
- **Tell me how the STOPS treatment approach differs from your usual physiotherapy treatment approach…?**

**Section 3: Perceptions on patient responses to STOPS physiotherapy treatment**

**Can you tell me about how patients with low back pain have been responding to your STOPS physiotherapy treatment in the trial?**

Prompts

- Tell me about any changes you have seen in patients, or changes they have told you about, in response to STOPS physiotherapy….?
- **Can you give me 1-2 examples of what changes have occurred in patients with the treatment?**

**Can you tell me what parts of the STOPS program contributed to the changes described above?**

Prompts:

- Which components of the STOPS physiotherapy program seemed to be leading to the changes described above…?
- Can you expand/tell me more about…?
- **Can you give me an example…??**

**Tell me about any feedback patients have been giving you about the STOPS physiotherapy treatment program?**

Prompts:

- What did they like or dislike about it…?
- Can you tell me about any difficulties or challenges patients have had with the treatment…?

**Section 4: Perceptions on barriers and facilitators to implementing STOPS physiotherapy treatment**

**Can you talk about what it was like for you delivering STOPS physiotherapy treatment for patients with low back pain in the trial?**

Prompts:

- Can you expand/tell me more about…?
- Are there any particular components of the STOPS treatment that come to mind when you think about your experiences delivering the physiotherapy treatment…?

**Can you tell me about any factors or circumstances that made it easier for you to implement STOPS physiotherapy in the trial?**

Prompts:

- Can you expand/tell me more about…?

**Tell me about *how* that made it easier for you…?**

**Can you tell me about any barriers or challenges to implementing the STOPS physiotherapy in the trial?**

Prompts:

- Can you expand/tell me more about…?
- **Expand on telling me about *how* that was a challenge for you…?**
- Did you manage to find a way to overcome that barrier…?
  - If so, how did you overcome it…?
  - If not, what might help to overcome that barrier…?

**Section 5: Future plans in relation to treating patients with LBP**

What are your plans for how you might treat LBP patients in the future?

- Can you talk about what approaches you are likely to use….?
- What factors influence that decision…?

**Section 6: Is there anything else you would like to say about STOPS physiotherapy that we have not covered in the interview?**

**Appendix 4- fMRI Protocol**

Machine name: Philips ingenia 3 Tesla R5

**Data acquisition**

Functional and structural (T1 EMPIRAGE) images will be acquired for each participant. The protocol for the data acquisition (Philips ingenia 3T specific) will be adopted from Alzheimer’s Disease Neuroimaging Initiative (ADNI) 3 protocol (1). The scanning duration will be kept as more than 10 minutes to ensure the reliability.

**Data Preprocessing**

Data will be preprocessed and analyzed using SPM12 (the welcome department of cognitive neurology, London, UK, <http://www.fil.ion.ucl.ac.uk/spm/software/spm12/>). The preprocessing pipeline used will be the standard conventional preprocessing pipeline in using the CONN-fMRI functional connectivity toolbox (http://www.nitrc.org/projects/conn).

All functional images will be slice-time corrected and realigned to the first volume using a six-parameter rigid body transformation. The anatomical image and functional images will be coregistered for the corresponding time-point. Segmented gray matter and white matter images of all participants will be used to construct a tissue probability maps. The template will be normalized to Montreal Neurological Institute (MNI) space and all images, anatomical and functional, will be normalized to this template using the according flow fields. The smoothing kernel for the functional images will be kept as 6 mm and 2 mm for the anatomical image. 2.5.

**Connectivity Analysis**

Functional connectivity analyses will be carried out using the CONN-fMRI functional connectivity toolbox v14. Seed-to-voxel and ROI-to-ROI functional connectivity maps will be created for each participant. The ROI-to-ROI analysis will be used to identify possible differences between before and after treatment. For this analysis we will use all the provided areas. The mean BOLD time series will be computed across all voxels within each ROI.

Individual seed-to-voxel and ROI-to-ROI maps will be entered into a second-level analysis. A within group ROI-to-ROI analysis will be performed. Seed-to-voxel analyses will be as necessary.

1. ADNI. MRI scanner protocols 2022 [Available from: <https://adni.loni.usc.edu/methods/documents/mri-protocols/>.

**APPENDIX 5: INFORMED CONSENT FORM**

**Project title**: Individualized physiotherapy versus usual care (STOPS trial) for Low Back Disorder in Indian primary care: A Randomized Clinical Trial

**Name and age of the participant**:

DOB of the participant:

1. I understand that I am being invited to take part in the research study. I confirm that I have read/ been read to and understood the information sheet dated _________ for the above study and have had the opportunity to ask questions.

2. I understand that my participation in the study is voluntary and that I am free to withdraw at any time, without giving any reason, without my medical care or legal rights being affected.

3. I understand the risks and potential benefits of this research study that were explained to me. I freely give my consent to take part in research study described in this form.

4. I understand the research study, IEC and the regulatory authorities will not need my permission to look at my health records both in respect of the current study and any further research that may be conducted in relation to it, even if I withdraw from the trial. I agree to this access. However, I understand that my identity will not be revealed in any information released to third parties or published.

5. I agree not to restrict the use of any data or results that arise from this study provided such a use is only for scientific purpose(s).

6. I agree to take part in the above study.

I have read/have been read the above information and agreed to participate in this study. I

have received a copy of this form.

| Participant’s name (print): |  |
| --- | --- |
| Participant’ss Signature/Thumb impression &date: |  |
| Address |  |
| Phone Nos: |  |
| Legal Acceptable Representative name |  |
| Legal Acceptable Representative  Signature/Thumb impression & date  (if applicable): |  |
| Address (capital letters):  Phone Nos: |  |
| Impartial Witness’s name : |  |
| Impartial Witness’s signature & date (if  applicable): |  |
| Address (capital letters): |  |
| Phone Nos: |  |
| Name of PI or Co-PI/Co-I: |  |
| PI or Co-PI/Co-I sign & date: |  |
