# Supplementary material for: STOPS approach to individualised physiotherapy versus usual physiotherapy care for chronic low back pain in India: A randomised controlled trial protocol
Source: PLoS One. 2025 Dec 30;20(12):e0339280. doi: 10.1371/journal.pone.0339280 (PMC12752971; doi:10.1371/journal.pone.0339280)
Supplement: S3 File — (DOCX) [file pone.0339280.s003.docx]

**S3 File: Description of the STOPS subgroups**

| **Classification into subgroups** |
| --- |
| 1. **Zygapophyseal joint dysfunction** |
| This group will comprise participants believed to have zygapophyseal joint dysfunction as the primary source of their back symptoms. To be classified in this subgroup, participants will have at least 3 of the following 4 features of zygapophyseal joint dysfunction:   1. presence of unilateral low back pain, 2. a regular compression pattern (pain reproduced with lumbar extension and ipsilateral lateral-flexion movements), 3. localized pain on ipsilateral passive postero-anterior accessory movement applied through the transverse process or the zygapophyseal joint at one or two segments and, 4. improvement in pain or range-of-movement following a “mini-treatment” of manual therapy directed at the zygapophyseal joint (1). |
| 1. **Reducible discogenic pain** |
| To be identified in this subgroup, participants will present with multiple features indicative of discogenic low back pain. The features have been identified through literature searching, consideration of causal mechanisms and the results of a Delphi study of experts in the field (2). The features are:   1. presence of low back pain with or without leg pain 2. sitting limited to less than 60 minutes 3. symptoms worse the next morning or the next day following the initial injury 4. history of working in a job involving manual handling 5. a mechanism of injury associated with flexion/rotation and/or compression loading 6. at least some difficulty with forward bending 7. at least some difficulty with lifting 8. at least some difficulty with sit-to-stand 9. at least some difficulty with coughing/sneezing.   Participants will also demonstrate a directional preference in response to mechanical loading strategies on physical examination. The presence of a directional preference has been proposed as identifying people likely to have discogenic pain where a posterior or posterolaterally migrated nucleus pulposus can be “reduced” into a more central and non-pain-provoking position (3-5). |
| 1. **Non-reducible discogenic pain** |
| To be identified in this subgroup, participants will have several signs of discogenic pain as outlined above for the reducible discogenic pain subgroup. However, participants who do not demonstrate a directional preference in response to mechanical loading strategies will be classified in the non-reducible (rather than reducible) discogenic pain subgroup provided they do not satisfy the selection criteria for the zygapophyseal joint dysfunction group [6]. Other features that may increase confidence in this diagnosis include:   - History of traumatic injury to the lumbar spine - History of heavy manual handling - Clinical features indicative of inflammation (6) - Pain worse with static standing (relative to sitting or walking) - Poor response to previous physical therapy treatments (eg. manual therapy or exercise) |
| 1. **Other nociceptive pain disorder** |
| Participants with a predominant nociceptive pain type who do not fit one of the above subgroups will be classified as belonging to this subgroup. For example, participants with sacroiliac joint dysfunction as defined by the following criteria:   - 1. Localised pain around the posterior superior iliac spine or sacroiliac joint (7).   2. History of trauma (e.g. a fall on the hip or buttock) or pregnancy-related pain onset.   3. Pain with load-bearing, particularly asymmetrically (e.g. stairs, getting out of a car)   4. Positive physical examination findings on some or all of the following sacroiliac joint tests:  1. distraction, 2. compression, 3. thigh thrust, 4. Gaenslen’s test, or sacral thrust (8). |
| 1. **Radicular disorders** |
| This subgroup defines participants with low back-related leg symptoms who meet the definition of neuropathic pain (9-11). This includes radicular pain or radiculopathy. |
| 1. **Predominant nociplastic pain** |
| Participants will be categorised to this subgroup if they are presumed to have a predominant nociplastic pain mechanism based on accepted criteria (9-13). |

**References**

1. Ford JJ, Thompson SL, Hahne AJ. A classification and treatment protocol for low back disorders: Part 1 – specific manual therapy. Physical Therapy Reviews. 2011;16(3):168-77.

2. Chan AY, Ford JJ, McMeeken JM, Wilde VE. Preliminary evidence for the features of non-reducible discogenic low back pain: survey of an international physiotherapy expert panel with the Delphi technique. Physiotherapy. 2013;99(3):212-20.

3. Petersen T, Laslett M, Thorsen H, Manniche C, Ekdahl C, Jacobsen S. Diagnostic classification of non-specific low back pain. A new system integrating patho-anatomic and clinical categories. Physiotherapy Theory and Practice. 2003;19(4):213-37.

4. Wetzel FT, Donelson R. The role of repeated end-range/pain response assessment in the management of symptomatic lumbar discs. Spine J. 2003;3(2):146-54.

5. Vining R, Potocki E, Seidman M, Morgenthal AP. An evidence-based diagnostic classification system for low back pain. J Can Chiropr Assoc. 2013;57(3):189-204.

6. Ford JJ, Kaddour O, Gonzales M, Page P, Hahne AJ. Clinical features as predictors of histologically confirmed inflammation in patients with lumbar disc herniation with associated radiculopathy. BMC Musculoskelet Disord. 2020;21(1):567.

7. Han CS, Hancock MJ, Sharma S, Sharma S, Harris IA, Cohen SP, et al. Low back pain of disc, sacroiliac joint, or facet joint origin: a diagnostic accuracy systematic review. EClinicalMedicine. 2023;59:101960.

8. Petersen T, Laslett M, Juhl C. Clinical classification in low back pain: best-evidence diagnostic rules based on systematic reviews. BMC Musculoskelet Disord. 2017;18(1):188.

9. Shraim MA, Massé-Alarie H, Hodges PW. Methods to discriminate between mechanism-based categories of pain experienced in the musculoskeletal system: a systematic review. Pain. 2021;162(4):1007-37.

10. Shraim MA, Sluka KA, Sterling M, Arendt-Nielsen L, Argoff C, Bagraith KS, et al. Features and methods to discriminate between mechanism-based categories of pain experienced in the musculoskeletal system: a Delphi expert consensus study. Pain. 2022;163(9):1812-28.

11. Kosek E, Clauw D, Nijs J, Baron R, Gilron I, Harris RE, et al. Chronic nociplastic pain affecting the musculoskeletal system: clinical criteria and grading system. Pain. 2021;162(11):2629-34.

12. Nijs J, Kosek E, Chiarotto A, Cook C, Danneels LA, Fernández-de-Las-Peñas C, et al. Nociceptive, neuropathic, or nociplastic low back pain? The low back pain phenotyping (BACPAP) consortium's international and multidisciplinary consensus recommendations. Lancet Rheumatol. 2024;6(3):e178-e88.

13. Hodges PW, Sanchez R, Pritchard S, Turnbull A, Hahne A, Ford J. Toward Validation of Clinical Measures to Discriminate Between Nociceptive, Neuropathic, and Nociplastic Pain: Cluster Analysis of a Cohort With Chronic Musculoskeletal Pain. Clin J Pain. 2025;41(5).
