# Supplementary material for: STOPS approach to individualised physiotherapy versus usual physiotherapy care for chronic low back pain in India: A randomised controlled trial protocol
Source: PLoS One. 2025 Dec 30;20(12):e0339280. doi: 10.1371/journal.pone.0339280 (PMC12752971; doi:10.1371/journal.pone.0339280)
Supplement: S4 File — (DOCX) [file pone.0339280.s004.docx]

**S4 File: Detailed description of STOPS treatment strategies**

1. ***Advice and education***

Throughout the physiotherapy sessions, participants will be provided with education and advice regarding the nature and management of their condition. This will include an explanation of pain type (ie nociceptive, neuropathic or nociplastic dominant pain) and if relevant, explanation of the pathoanatomical diagnosis. Information will also be provided regarding prognosis, treatment options, and the proposed mechanisms of treatment effect (1).

1. ***Goal setting***

Participants in collaboration with the physiotherapist will develop meaningful goals (2) that will direct a graded exercise and activity program. At the beginning of the program, participants will be asked to identify short, medium and long-term goals regarding activities (e.g., hobbies or work) or other aspects of their lives such as relationships or general health. The participant and physiotherapist will then develop a graded activity and exercise program that specifically targets the achievement of these goals (1).

1. ***Pacing***

Participants may be taught to pace their activities appropriately to avoid exacerbations of their pain due to over or under-activity. This involves supporting participants to find a baseline tolerance for a particular activity, and then initially limiting the activity below that threshold until tolerance has improved. In patients with a primary nociceptive or neuropathic pain mechanism, effective pacing will be a focus prior to commencing graded activity and functional exercise (1). For patients with a nociplastic dominant pain type pacing strategies will still be explored as a pain management strategy, but with caution not to reinforce unhelpful beliefs on avoidance of activity.

1. ***Management of inflammation***

Participants with validated clinical signs of inflammation (3) in conjunction with a primary nociceptive or neuropathic pain mechanism will be encouraged to see their pharmacist or medical practitioner to discuss anti-inflammatory medication options. Regular walking, lumbar spine taping, and postural modification will also be implemented in an effort to control inflammatory processes. Management of inflammation will be a focus prior to commencing graded activity and exercise.

1. ***Sleep management***

Participants with disturbed sleep due to low back pain will be provided with sleep strategies, such as the promotion of a sleep routine, relaxation prior to bed, mindfulness, and appropriate bedding and positioning. Sleeping posture will be explored for patients with a nociceptive/neuropathic dominant pain type. Medication strategies for the management of sleep may also be discussed with the participant’s treating medical practitioner (1).

1. ***Motor control training***

In participants with a primary nociceptive or neuropathic pain mechanism, lumbo-pelvic motor control strategies will be assessed. In cases where unhelpful motor control is noted specific muscle activation of transversus abdominis and lumbar multifidus (4-6) will be undertaken. Where necessary this will incorporate relaxation and retraining of over-active muscles. Motor control training will generally commence in a lying position, progress to standing, and then be integrated into functional activities (such as walking, squatting, lifting and bending). Achieving adequate motor control in walking will be a focus prior to commencing graded activity and functional exercise.

Participants may be invited to alter their posture and movements in a way that is conducive to recovery from their particular injury (e.g., avoiding excessive lumbar flexion in the presence of a disc herniation with a predominant neuropathic pain pattern) or in a way that relieves their pain (e.g., maintaining straighter posture when sitting if this results in pain relief) (4). Movements and postures will be identified as either helpful or unhelpful based on immediate response to adjustment of the movement or posture.

1. ***Management of psychosocial barriers to recovery***

High-priority psychosocial barriers to recovery determined by physiotherapy assessment and baseline screening will be addressed using a variety of strategies (7) including:

- Cognitive restructuring of unhelpful thoughts or beliefs that are expressed by the participant in the baseline assessment and associated questionnaires (7, 8).
- Education relating to overcoming fear of movement and activity, by understanding that pain in response to gentle activity (such as short walks or mild bending) in persistent low back pain is not indicative of tissue damage, hence these activities do not need to be completely avoided (but perhaps modified or paced appropriately) (9).
- Some behavioural strategies will also be implemented, such as positive reinforcement of behaviour that is likely to improve recovery of function (eg. encouragement and praise is given to participants when they increase their exercises appropriately or achieve an activity goal) (10).

1. ***Specific manual therapy***

In participants where a clinical pattern is identified that is indicative of a likely positive response to manual therapy, specific manual therapy will be provided as described in the STOPS-specific manual therapy protocol (11).

1. ***Directional preference management***

In participants where a directional preference is identified, directional preference management (based on the McKenzie method) will be applied in accordance with the directional preference management protocol (12).

1. ***Graded exercise***

Participants will be instructed in a graded exercise program aiming to improve functional capacity to facilitate the achievement of the identified goals. These exercises will be performed in the clinic under the physiotherapist’s supervision as well as at home. Common exercises that may be suitable for participants (based on experience from our previous trial (13)) include: walking, stationary bike, step-ups, bicep curls, forward raises, squats, lunges and lifting progressing to functionally relevant exercise individualized to the patient goals. The starting dosage for weights is generally low (0.5 to 2kg) and then progresses gradually depending on the participants' goals (eg. a participant with a goal of being able to lift their 15kg child will progress up to 15kg weights for lifting practice). Progressions will be negotiated with the participant and monitored closely by the physiotherapist to ensure correct exercise technique and to avoid unreasonable symptom exacerbation. Participants with a primary nociceptive or neuropathic pain mechanism will progress exercise in a pain-contingent manner (6), whereas those with a primary nociplastic pain mechanism will progress in a negotiated time-contingent manner (7).

1. ***Graded activity***

All participants will work collaboratively with the physiotherapist on a graded activity program to facilitate a progressive increase in activities that are limited due to the participant’s low back condition. Over time the participant will progress to targeting medium- and long-term activity goals thereby increasing engagement with meaningful activities and improving quality of life (6).

1. ***Management of pain***

Strategies for the management of daily pain will be recommended to participants. These options will include the self-application of ice or heat, and exercises that may relieve pain (such as gentle stretches). Participants will also be encouraged to follow the advice of their pharmacist and/or medical practitioner to ensure that adequate pain medication is available (1).

1. ***Mindfulness***

Mindfulness will be encouraged through an information sheet and direction to access an online mindfulness App (14).

1. ***Management of increases in pain***

Participants who report a persisting increase in their usual level of symptoms (eg. from an activity they performed at home) will be provided with strategies to manage their increased symptoms. The physiotherapist will help to identify the cause of the increase in pain to avoid repeat occurrences, posture and taping may need to be re-visited, and exercises may need to be temporarily modified until pain resides. For significant and persistent increases in pain, the physiotherapist will reassess the participant, and refer them to their medical practitioner for further investigation or management if necessary (1).

1. ***Referral to other healthcare providers***

If physiotherapists identify that a participant is not responding well to treatment or possesses barriers to recovery that might require the input of another healthcare practitioner (such as a psychologist if a patient becomes highly depressed), then a referral to their medical practitioner will be made for consideration of additional or alternative intervention (1).

**References**

1. Hahne AJ, Ford JJ, Surkitt LD, Richards MC, Chan AY, Thompson SL, et al. Specific treatment of problems of the spine (STOPS): design of a randomised controlled trial comparing specific physiotherapy versus advice for people with subacute low back disorders. BMC Musculoskelet Disord. 2011;12:104.

2. Sowden M, Hatch A, Gray SE, Coombs J. Can four key psychosocial risk factors for chronic pain and disability (Yellow Flags) be modified by a pain management programme?: A pilot study. Physiotherapy. 2006;92(1):43-9.

3. Ford JJ, Kaddour O, Gonzales M, Page P, Hahne AJ. Clinical features as predictors of histologically confirmed inflammation in patients with lumbar disc herniation with associated radiculopathy. BMC Musculoskelet Disord. 2020;21(1):567.

4. Hodges P, Van Dillen L, McGill S, Brumange S, Hides J, Moseley GL. Integrated clinical approach to motor control interventions in low back and pelvic pain (Chapter 21). Spinal control: The rehabilitation of back pain State of the art and science: Churchill Livingstone; 2013.

5. Ford JJ, Bower SE, Ford I, De Mello MM, Carneiro SR, Balasundaram AP, et al. Effects of specific muscle activation for low back pain on activity limitation, pain, work participation, or recurrence: A systematic review. Musculoskeletal Science and Practice. 2020;50:102276.

6. Ford JJ, Hahne AJ, Chan AYP, Surkitt LD. A classification and treatment protocol for low back disorders: Part 3- Functional restoration for intervertebral disc related problems. Phys Ther Rev. 2012;17(1):55-75.

7. Ford JJ, Richards MC, Hahne AJ. A classification and treatment protocol for low back disorders: Part 4- Functional restoration for multi-factorial persistent pain. Phys Ther Rev. 2012;17(5):322-34.

8. Foster NE, Delitto A. Embedding psychosocial perspectives within clinical management of low back pain: integration of psychosocially informed management principles into physical therapist practice--challenges and opportunities. Phys Ther. 2011;91(5):790-803.

9. Butler D, Moseley G. Explain Pain: Noigroup Publications; 2003.

10. Lindström I, Ohlund C, Eek C, Wallin L, Peterson LE, Fordyce WE, et al. The effect of graded activity on patients with subacute low back pain: a randomized prospective clinical study with an operant-conditioning behavioral approach. Phys Ther. 1992;72(4):279-90; discussion 91-3.

11. Ford JJ, Richards MC, Hahne AJ. A classification and treatment protocol for low back disorders. Physical Therapy Reviews. 2012;17(5):322-34.

12. Ford JJ, Surkitt LD, Hahne AJ. A classification and treatment protocol for low back disorders: Part 2- Directional preference management for reducible discogenic pain. Phys Ther Rev. 2011;16(6):423-37.

13. Ford JJ, Hahne AJ, Surkitt LD, Chan AY, Richards MC, Slater SL, et al. Individualised physiotherapy as an adjunct to guideline-based advice for low back disorders in primary care: a randomised controlled trial. British journal of sports medicine. 2016;50(4):237-45.

14. App TM. Nurture A Healthy State Of Mind 2025 [Available from: <https://www.themindfulnessapp.com/>.
