# Supplementary material for: STOPS approach to individualised physiotherapy versus usual physiotherapy care for chronic low back pain in India: A randomised controlled trial protocol
Source: PLoS One. 2025 Dec 30;20(12):e0339280. doi: 10.1371/journal.pone.0339280 (PMC12752971; doi:10.1371/journal.pone.0339280)
Supplement: S5 File — (DOCX) [file pone.0339280.s005.docx]

**S5 File: Description of secondary outcome measures**

1. *Back pain and leg pain intensity:* Separate 0-10 Numerical Pain Rating Scales will be administered, one for back pain and another for leg pain. The intensity of pain will be rated on average over the past week, with end-point descriptors of “no pain” and “worst pain possible” (1).
2. *Pain severity and Interference:* Brief Pain Inventory - Short Form (BPI-SF) will be used to assess the severity of pain and the extent to which pain interferes with daily activities. The BPI-SF is a 9-item self-administered questionnaire which exists within the biopsychosocial model of pain, as it addresses sensory, emotional, and functional aspects of the pain experience (2). The tool is responsive to changes in pain associated with pharmacological, physical, and psychological interventions (3).
3. *Global effect:* Global rating of change will be measured using a 7-point Likert scale, with participants rating their overall change since the baseline assessment as “completely recovered”, “much improved”, “slightly improved”, “no change”, “slightly worsened”, “much worsened”, or “vastly worsened” (4). Various versions of this scale are reliable, responsive, and valid (4, 5).
4. *Satisfaction with treatment:* Participants will rate their satisfaction with physiotherapy treatment on a 5-point Likert scale, with ratings from “very satisfied” to “very dissatisfied” (6). This scale has good reliability, validity, and responsiveness (6, 7).
5. *Work interference:* Interference with work due to the LBP will be assessed in two ways. Firstly, at each assessment point, participants will record the number of work hours missed due to their back/leg condition over the previous 7 days (8, 9). Secondly, participants will rate the degree of interference with work productivity attributed to their back/leg condition over the previous week (8, 9).
6. *Psychosocial risk factors:* The Short-Form Örebro Musculoskeletal Pain Screening Questionnaire (ÖMPSQ-SF) will be used as a measure of psychosocial risk factors for chronic musculoskeletal pain (10, 11). Although it is more commonly used as a prognostic screening tool at one point in time (12), in our trial the Orebro will also be administered at each follow-up point to detect changes in psychosocial risk factors over time (13).
7. *Health-related quality of life:* Health-related quality of life will be measured with the EuoQol-5D-5L (14). The EuroQol-5D-5L has good reliability, validity, and responsiveness (15, 16).
8. *Mental health symptoms:* Mental health symptoms will be assessed using the 21-item Depression Anxiety and Stress Scale (DASS-21). The questionnaire measures the severity of depression, anxiety, and stress from normal to extremely severe. DASS-21 shows strong validity, test-retest reliability, internal consistency, and responsiveness (17).
9. *Pain self-efficacy:*  Pain self-efficacy will be assessed using the Pain Self-Efficacy Questionnaire (PSEQ). The PSEQ is a 10-item scale with a score ranging from 0 to 60, where high scores indicate greater levels of confidence in dealing with pain (18). The PSEQ has excellent validity, reliability, and responsiveness (19).
10. *Pain catastrophising:* The 13-item Pain Catastrophizing Scale (PCS) will be used to assess inappropriate coping strategies and catastrophic thinking about LBP. The scale is rated on a 5-point Likert scale ranging from 0 (not at all) to 4 (all the time). Total scores range from 0 to 52, with higher scores indicating worse catastrophic thinking about LBP. The PCS has been shown to have adequate validity, reliability and responsiveness (20-22).
11. *Sleep quality:* The Insomnia Severity Index (ISI) will be used to assess sleep quality. The scale comprises 7 questions that assess current sleep problems that are rated on a 5-point Likert scale (‘0’ representing none or not at all and ‘4’ representing very much). Total scores range from 0 to 28, with higher scores indicating worse insomnia severity. The ISI demonstrated good validity and responsiveness and has been used to assess sleep quality in individuals with LBP (23, 24).
12. *Central sensitisation:* The Central Sensitisation Inventory (CSI) will be used to assess central sensitization symptoms. The CSI is a 25-point scale, rated on a scale of 0 (never) to 4 (always), with a score of more than 40 indicating the presence of central sensitization. The CSI is a useful and valid measure for screening patients with central sensitization symptoms and has strong psychometric properties (25, 26).
13. *Inflammatory symptoms:* The 4-item clinical inflammation score will be used to assess low back-related inflammatory symptoms (27). A positive clinical inflammation score of at least 3 of; constant symptoms, morning pain/stiffness greater than 60-min, short walking not easing symptoms and significant night symptoms will be used to inform clinical decision. This scale has been validated and achieved a sensitivity of 90.9%, a specificity of 92.9%, and predictive accuracy of 92.3% (27).
14. *Treatment acceptability:* The treatment Credibility Questionnaire will be used to determine the acceptability of each treatment approach (28). Treatment acceptability will be assessed at each follow-up visit via a 4-item questionnaire rated on a scale of 0-10, at this point, how logical does the treatment offered to you seem? At this point, how successful do you think this treatment will be in helping you with your back problem? How confident would you be in recommending this treatment to a friend who experiences similar problems? By the end of your treatment, how much improvement in your back condition do you think will occur? This questionnaire demonstrated high internal consistency and good test-retest reliability and has been used in other LBP trials (29).
15. *Healthcare utilization:* A patient diary will be used to track healthcare utilization including imaging, medication, and other healthcare services (30).

**References**

1. Hartrick CT, Kovan JP, Shapiro S. The numeric rating scale for clinical pain measurement: a ratio measure? Pain pract. 2003;3(4):310-6.

2. Tan G, Jensen MP, Thornby JI, Shanti BF. Validation of the Brief Pain Inventory for chronic nonmalignant pain. J Pain. 2004;5(2):133-7.

3. Mendoza T, Mayne T, Rublee D, Cleeland C. Reliability and validity of a modified Brief Pain Inventory short form in patients with osteoarthritis. Eur J Pain. 2006;10(4):353-61.

4. Kamper S. Global Rating of Change scales. Aust J Physiother. 2009;55(4):289.

5. Kamper SJ, Maher CG, Mackay G. Global rating of change scales: a review of strengths and weaknesses and considerations for design. J Man Manip Ther. 2009;17(3):163-70.

6. Hudak PL, Wright JG. The characteristics of patient satisfaction measures. Spine (Phila Pa 1976). 2000;25(24):3167-77.

7. Ferrer M, Pellise F, Escudero O, Alvarez L, Pont A, Alonso J, et al. Validation of a minimum outcome core set in the evaluation of patients with back pain. Spine. 2006;31(12):1372-9; discussion 80.

8. Ford JJ, Hahne AJ, Surkitt LD, Chan AY, Richards MC, Slater SL, et al. Individualised physiotherapy as an adjunct to guideline-based advice for low back disorders in primary care: a randomised controlled trial. Br J Sports Med. 2016;50(4):237-45.

9. Hahne AJ, Ford JJ, Surkitt LD, Richards MC, Chan AY, Thompson SL, et al. Specific treatment of problems of the spine (STOPS): design of a randomised controlled trial comparing specific physiotherapy versus advice for people with subacute low back disorders. BMC Musculoskelet Disord. 2011;12:104.

10. Linton SJ, Boersma K. Early identification of patients at risk of developing a persistent back problem: the predictive validity of the Orebro Musculoskeletal Pain Questionnaire. Clin J Pain. 2003;19(2):80-6.

11. Linton SJ, Hallden K. Can we screen for problematic back pain? A screening questionnaire for predicting outcome in acute and subacute back pain. Clin J Pain. 1998;14(3):209-15.

12. Hockings RL, McAuley JH, Maher CG. A systematic review of the predictive ability of the Orebro Musculoskeletal Pain Questionnaire. Spine (Phila Pa 1976). 2008;33(15):E494-500.

13. Hovens C, Ford J, Hahne A. Minimum clinically important difference of the original and short-form Örebro musculoskeletal pain questionnaire for low back pain. Musculoskelet Sci Pract. 2025;78:103352.

14. EuroQOL-Group. EuroQol--a new facility for the measurement of health-related quality of life. Health Policy. 1990;16(3):199-208.

15. Hurst NP, Kind P, Ruta D, Hunter M, Stubbings A. Measuring health-related quality of life in rheumatoid arthritis: validity, responsiveness and reliability of EuroQol (EQ-5D). Br J Rheumatol. 1997;36(5):551-9.

16. Linde L, Sorensen J, Ostergaard M, Horslev-Petersen K, Hetland ML. Health-related quality of life: validity, reliability, and responsiveness of SF-36, 15D, EQ-5D [corrected] RAQoL, and HAQ in patients with rheumatoid arthritis. J Rheumatol. 2008;35(8):1528-37.

17. Lee D. The convergent, discriminant, and nomological validity of the Depression Anxiety Stress Scales-21 (DASS-21). J Affect Disord. 2019;259:136-42.

18. Nicholas MK. The pain self-efficacy questionnaire: Taking pain into account. Eur J Pain. 2007;11(2):153-63.

19. Dubé MO, Langevin P, Roy JS. Measurement properties of the Pain Self-Efficacy Questionnaire in populations with musculoskeletal disorders: a systematic review. Pain Rep. 2021;6(4):e972.

20. Franchignoni F, Giordano A, Ferriero G, Monticone M. Measurement precision of the Pain Catastrophizing Scale and its short forms in chronic low back pain. Sci Rep. 2022;12(1):12042.

21. Cook KF, Mackey S, Jung C, Darnall BD. The factor structure and subscale properties of the pain catastrophizing scale: are there differences in the distinctions? Pain Rep. 2021;6(1):e909.

22. Osman A, Barrios FX, Kopper BA, Hauptmann W, Jones J, O'Neill E. Factor structure, reliability, and validity of the Pain Catastrophizing Scale. Journal of behavioral medicine. 1997;20(6):589-605.

23. Bastien CH, Vallières A, Morin CM. Validation of the Insomnia Severity Index as an outcome measure for insomnia research. Sleep Med. 2001;2(4):297-307.

24. Alsaadi SM, McAuley JH, Hush JM, Bartlett DJ, McKeough ZM, Grunstein RR, et al. Assessing sleep disturbance in low back pain: the validity of portable instruments. PLoS One. 2014;9(4):e95824.

25. Scerbo T, Colasurdo J, Dunn S, Unger J, Nijs J, Cook C. Measurement properties of the central sensitization inventory: a systematic review. Pain pract. 2018;18(4):544-54.

26. Neblett R. The central sensitization inventory: a user’s manual. Journal of Applied Biobehavioral Research. 2018;23(2):e12123.

27. Ford JJ, Kaddour O, Gonzales M, Page P, Hahne AJ. Clinical features as predictors of histologically confirmed inflammation in patients with lumbar disc herniation with associated radiculopathy. BMC Musculoskeletal Disorders. 2020;21(1).

28. Devilly GJ, Borkovec TD. Psychometric properties of the credibility/expectancy questionnaire. J Behav Ther Exp Psychiatry. 2000;31(2):73-86.

29. Smeets RJ, Beelen S, Goossens ME, Schouten EG, Knottnerus JA, Vlaeyen JW. Treatment expectancy and credibility are associated with the outcome of both physical and cognitive-behavioral treatment in chronic low back pain. Clin J Pain. 2008;24(4):305-15.

30. Hahne AJ, Ford JJ, Surkitt LD, Richards MC, Chan AY, Slater SL, et al. Individualized Physical Therapy is Cost Effective Compared to Guideline-Based Advice for People with Low Back Disorders. Spine (Phila Pa 1976). 2017;42(3):E169-E76.
