# Supplementary material for: STOPS approach to individualised physiotherapy versus usual physiotherapy care for chronic low back pain in India: A randomised controlled trial protocol
Source: PLoS One. 2025 Dec 30;20(12):e0339280. doi: 10.1371/journal.pone.0339280 (PMC12752971; doi:10.1371/journal.pone.0339280)
Supplement: S6 File — (DOCX) [file pone.0339280.s006.docx]

**S6 File: Interview guide for patients**

*Aims*

1. Explore participants’ thoughts and feelings around **their experiences** throughout the 11-week physiotherapy program for their low back pain
2. Compare and contrast perceptions about the current physiotherapy program relative to previous treatments received (if any), and relative to expectations coming in to treatment.
3. Explore participants’ thoughts and feelings about **changes** that they experienced throughout the 11-week treatment program
4. Explore participants’ expectations on their **plans for the future** for managing their low back pain.

**Brief introduction**

Thankyou for participating in the research study. We ask some participants at the end of their treatment to share their thoughts and experiences about the treatment they just received.

**Section 1: Perceptions of the program**

**What are your thoughts about the physiotherapy program you’ve just completed?**

**Prompts:**

- Can you expand/tell me more about…?
- Can you talk about what the experience was like for you completing the physiotherapy treatment program….?
- Can you elaborate on any particular aspects of the physiotherapy program that come to mind……?

**Can you tell me about some of things that you and your physiotherapist talked about regarding your back pain?**

**Prompts:**

- And what are your thoughts about that…..
- Is there anything else that you recall talking about in relation to your back pain…
- Can you tell me about anything you learnt about managing your back pain….

**Section 2: Perceptions on the treatment relative to previous treatment or expectations**

**Can you tell me about other treatments you have tried previously for your low back condition?**

**Prompts (esp. if previous physiotherapy, but also other treatments)**

- How did that treatment compare to the physiotherapy treatment you just received….?

**Coming into the physiotherapy treatment program, what were your expectations?**

**Prompts**

- And how did the program you just completed match up with those expectations…..?
- What were you expecting the physiotherapy would involve….?

**Section 3: Perceptions on changes from the program**

**Can you talk about how you feel now compared to before the treatment program?**

**Prompts**

- Can you expand/tell me more about…?
- (if they mention being better or worse) – in what ways are you better / worse, what has changed…?
- Can you tell me about 1 or 2 examples of what has changed…?

**How do you feel now about managing your low back condition? How does that compare to before treatment?**

**Prompts**

- Can you expand/tell me more about…

**How do you feel now about doing your usual activities? How does that compare to before treatment?**

**Prompts**

- Can you expand/tell me more about…? Can you give me an example of how 1 or 2 activities are now compared to before treatment?

**Can you tell me what parts of the treatment program contributed to the changes you described above?**

**Prompts:**

- Which parts of the physiotherapy program contributed most to the differences you described?
- Can you expand/tell me more about…?

**Section 4: Plans for the future**

**Can you tell me about your plans for managing your low back condition in the future**

**Prompts**

- Can you expand/tell me more about…?
- Are there be any other treatments that you are considering…?

**Section 5: Is there anything else you would like to say that we have not covered in the interview?**
