# Supplementary material for: STOPS approach to individualised physiotherapy versus usual physiotherapy care for chronic low back pain in India: A randomised controlled trial protocol
Source: PLoS One. 2025 Dec 30;20(12):e0339280. doi: 10.1371/journal.pone.0339280 (PMC12752971; doi:10.1371/journal.pone.0339280)
Supplement: S7 File — (DOCX) [file pone.0339280.s007.docx]

**S7 File: Procedures for fMRI**

Resting state and task based functional MRI (fMRI) data will be obtained for 15 individualised physiotherapy participants, 15 usual care participants, and 10 healthy controls. The data will be acquired using United Imaging 1.5 Tesla machine. Functional and structural (T1 EMPIRAGE) images will be acquired for each participant. The functional scanning parameters includes TR of 3000, flip angle of 90 degree, TE of 30 and slice thickness of 35 mm. The scanning duration for the resting state fMRI will be kept as more than 8 minutes to ensure the reliability (1).

All participants recruited for the fMRI study will undergo an eligibility screening specific to the MRI. This includes willingness and readiness for the fMRI scan, weight less than 110 kilograms, presence of claustrophobia (using the claustrophobia questionnaire), presence of any metal implants, pacemakers on the body, no history of neurological or psychiatric disorders, no history of brain surgeries (2). Followed by this, participants will undergo a familiarization session on a different day from the actual MRI session, outside the MRI machine to acquaint with the procedure. Homogeneity of the data will be facilitated by ensuring that both groups have an equal number of participants matched by age, pain type, LBP duration, pain intensity, and gender.

Prior to the fMRI (at both baseline and follow-up), all patients and healthy controls will be subjected to pain neurophysiology measures (Pressure pain thresholds, Two point discrimination distance and Mechanical temporal summation) (3, 4). Participants will firstly undergo a resting state fMRI scan. For the resting state fMRI, participants will be comfortably positioned supine in the scanner and instructed to lie still, keep their eyes open, and focus on a fixation point, allowing natural thoughts to flow without attempting to control them. Continuous monitoring will be provided via an intercom system, and visual or auditory cues will be used if necessary to remind participants to remain still.

For the task-based fMRI, a task involving repetitive active straight-leg raises is planned. Participants will lift and hold their leg in the air at 10-20 degrees of hip flexion for a 30 second period, followed by 30 seconds of rest (5). The hip movement will be a lower force movement, to avoid any unintended movement along with the target movement. This will be repeated for 5 bouts of alternative leg lifting and rest periods. The leg raises will be performed on their most symptomatic side, or on their side of hand dominance for central LBP or where pain (measured using NPRS scale) is equal on both sides. During the task-based fMRI session, participants will receive verbal cues every 30 seconds to initiate and lower each leg raise to maintain correct timing. The schematic representation of the procedure is pictured below.

**References**

1. Birn RM, Molloy EK, Patriat R, Parker T, Meier TB, Kirk GR, et al. The effect of scan length on the reliability of resting-state fMRI connectivity estimates. Neuroimage. 2013;83:550-8.

2. Lv H, Wang Z, Tong E, Williams LM, Zaharchuk G, Zeineh M, et al. Resting-State Functional MRI: Everything That Nonexperts Have Always Wanted to Know. AJNR Am J Neuroradiol. 2018;39(8):1390-9.

3. Rolke R, Magerl W, Campbell KA, Schalber C, Caspari S, Birklein F, et al. Quantitative sensory testing: a comprehensive protocol for clinical trials. Eur J Pain. 2006;10(1):77-88.

4. Georgopoulos V, Akin-Akinyosoye K, Zhang W, McWilliams DF, Hendrick P, Walsh DA. Quantitative sensory testing and predicting outcomes for musculoskeletal pain, disability, and negative affect: a systematic review and meta-analysis. Pain. 2019;160(9):1920-32.

5. James JS, Rajesh P, Chandran AV, Kesavadas C. fMRI paradigm designing and post-processing tools. Indian J Radiol Imaging. 2014;24(1):13-21.
