# Supplementary material for: STOPS approach to individualised physiotherapy versus usual physiotherapy care for chronic low back pain in India: A randomised controlled trial protocol
Source: PLoS One. 2025 Dec 30;20(12):e0339280. doi: 10.1371/journal.pone.0339280 (PMC12752971; doi:10.1371/journal.pone.0339280)
Supplement: Attachment — Submitted filename: Response to reviewers.docx [file pone.0339280.s008.docx]

PONE-D-25-44126

STOPS approach to individualised physiotherapy versus usual physiotherapy care for chronic low back pain in India: A randomised controlled trial protocol

**Editor comments / Journal Requirements:**

When submitting your revision, we need you to address these additional requirements.

1. Please ensure that your manuscript meets PLOS ONE's style requirements, including those for file naming. The PLOS ONE style templates can be found at

<https://journals.plos.org/plosone/s/file?id=wjVg/PLOSOne_formatting_sample_main_body.pdf> and 
<https://journals.plos.org/plosone/s/file?id=ba62/PLOSOne_formatting_sample_title_authors_affiliations.pdf>

**Response: The style requirements have been cross-checked.**

2. We noticed you have some minor occurrence of overlapping text with the following previous publication(s), which needs to be addressed:

<https://www.sciencedirect.com/science/article/abs/pii/S1551714425001545?via%3Dihub>

In your revision ensure you cite all your sources (including your own works), and quote or rephrase any duplicated text outside the methods section. Further consideration is dependent on these concerns being addressed.

**Response: The first author of the RCT protocol submitted to PLOS One is the last (senior) author of another clinical trial being conducted by the same research team in Bangladesh. There are overlapping methods and statistical analysis plans for these trials, however care has been taken to minimise overlap in text, especially in the introduction and discussion sections. In addition, the Bangladesh trial is not a RCT, whereas the current submission to PLOS One is an RCT, hence several methods differ.**

3. Thank you for stating the following financial disclosure:

“JSS College of Physiotherapy funded the fMRI scans and physiotherapy treatment.”

Please state what role the funders took in the study.  If the funders had no role, please state: "The funders had no role in study design, data collection and analysis, decision to publish, or preparation of the manuscript." If this statement is not correct you must amend it as needed.

Please include this amended Role of Funder statement in your cover letter; we will change the online submission form on your behalf.

**Response: The following financial disclosure has been adjusted in the cover letter:**

**“JSS College of Physiotherapy funded the fMRI scans and physiotherapy treatment. The funders had no role in study design, data collection and analysis, decision to publish, or preparation of the manuscript”.**

4. Please note that funding information should not appear in the Acknowledgments section or other areas of your manuscript. We will only publish funding information present in the Funding Statement section of the online submission form. Please remove any funding-related text from the manuscript.

**Response: The financial disclosure on the title page of the original submission has now been removed.**

5. If the reviewer comments include a recommendation to cite specific previously published works, please review and evaluate these publications to determine whether they are relevant and should be cited. There is no requirement to cite these works unless the editor has indicated otherwise.

**Response: No additional citations have been requested or added.**

6. Please review your reference list to ensure that it is complete and correct. If you have cited papers that have been retracted, please include the rationale for doing so in the manuscript text, or remove these references and replace them with relevant current references. Any changes to the reference list should be mentioned in the rebuttal letter that accompanies your revised manuscript. If you need to cite a retracted article, indicate the article’s retracted status in the References list and also include a citation and full reference for the retraction notice.

**Response: The reference list has been reviewed and no changes were necessary.**

**Reviewers' comments:**

**Reviewer #1: Comments**

General comments: This is a well-written protocol that largely follows rigorous statistical analytic techniques for assessing the effectiveness of STOPS. The cost-effectiveness analysis and qualitative aspect are quite detailed and well written. Here are a few comments

1. State the type of trial being conducted (superiority, non-inferiority, equivalence)

**Response: Thank-you for this suggestion, it has been addressed to confirm to readers that we are conducting a superiority RCT.**

**Action: The following text has been modified:**

**Abstract *“This is a parallel-group superiority randomised controlled trial…” (Line 37)***

**Study design *“This participant, outcome assessor, and data analyst-blinded, parallel-group superiority RCT will examine the effectiveness of individualised physiotherapy based on the STOPS approach versus standard physiotherapy (usual care) for the management of CLBP in India”.* (Line 93)**

2. Clearly state the hypothesis the hypothesis to be tested based on the type of trial being conducted

**Response: We have indicated our hypothesis to the revised manuscript based on this helpful suggestion.**

**Action: The following text has been added to the end of the introduction, immediately after the aim:**

***“It is hypothesised that individualised physiotherapy (STOPS) will produce superior outcomes to usual physiotherapy care”.* (Lines 88-89)**

3. The authors indicated that all participants would receive 10 x 45-minute physiotherapy sessions throughout 10 weeks, but did not provide any justification for the intervention period and its implications. Why 10 weeks? For instance, does extending the period beyond 10 weeks change the effect of the intervention (positive or negative)? What would have been the implication (positive or negative) if a shorter duration had been chosen? Details must be provided

**Response: The 10-week intervention period was chosen to match the original STOPS trial evaluated in Australia and found to be effective. One additional booster session was added for the current trial in an attempt to promote sustained treatment effects in a chronic LBP population.**

**Action: The text has been modified as indicated below.**

***“All participants will receive 10 x 45-minute physiotherapy sessions throughout a 10-week period, matching the period of intervention in the successful STOPS trial in Australia (13). An 11^th^ session will occur 6-months post randomisation to review, reinforce and progress the treatment strategies”.* (Line 192)**

4. Separate the power analysis (sample size calculations) from the data analysis section. There should be separate subheadings for sample size (power analysis) and statistical analysis to aid the flow of the protocol. The sample size estimation must be based on a clearly defined hypothesis and the type of RCT, as stated previously. There should be a section on data quality assurance for the trial.

**Response: Thank-you for these suggestions. They have been actioned as outlined below:**

**Action: The power analysis has been moved to a separate heading labelled “sample size”, with the hypothesis and type of trial now added to this section for context as indicated below:**

***“This superiority RCT will test the null hypothesis that there is no difference in Oswestry scores between individualised physiotherapy and usual physiotherapy care”.* (Lines 341-342)**

**Action: Additional information relating to data quality assurance has now been incorporated under the “Data management and trial monitoring” subheading on page 18, with the below text edited:**

***“Study personnel will complete Good Clinical Practice training. Standardised questionnaires, assessment methods and recording methods will be employed to ensure consistency of data collection for all participants”.* (Lines 316-318)**

***“All data will be entered into password-protected computers of the researchers, along with scanned copies of paper-based questionnaires for auditing and archiving purposes. Regular random audits will be conducted to ensure accurate data entry consistent with the scanned questionnaires. Audits will also involve cross-checking and triangulation of data from different sources, such as between participant questionnaires and treating therapist clinical notes”.* (Lines 325-327)**

5. Kindly include the key assumptions under linear mixed-effects models and how these assumptions will be tested. Include regression modelling strategies that will be adopted if these assumptions are violated

**Response: Based on this helpful suggestion, the statistical analysis section has been expanded.**

**Action: The following text has been added to capture the assessment of key assumptions for using linear mixed-effects models.**

***“Key assumptions for using linear mixed-effects models including linearity, normally distributed residuals, and homoscedasticity, will be evaluated via residual plots (Q-Q plots, histograms) and scatter plots. Where assumptions are violated, data transformation methods (eg. log transformation) will be attempted, and if unsuccessful, non-parametric analysis methods will be used”.* (Lines 359-364)**

6. The authors stated that “For ordinal outcomes, the Mann-Whitney U test will be used,” but did not indicate what the test would be used for. The statement is not complete

**Response: Thank-you for identifying this issue. It has been addressed as indicated below.**

**Action: The text has been expanded as shown below.**

***“For ordinal outcomes (eg. Global Rating of Change, Satisfaction with treatment, Clinical Inflammation Score), the Mann-Whitney U test will be used to determine whether there are statistically significant differences in medians between groups”.* (Lines 365-367)**

7. The statement “Dichotomous outcomes will be presented using risk ratios and the number needed to treat (with associated 95% confidence intervals), with significance determined using chi-square (χ²) analyses.” is unclear and somewhat confusing. Please revise and specify when you will use the Chi-square test and break sentences to make it a bit clearer.

**Response: Thank-you for identifying this. It has been addressed by relocating the text immediately below the analysis it relates to, as well as rephrasing. In addition, as hypothesis testing is not planned for this additional analysis, the Chi square test will not be required (hence this has been deleted).**

**Action: The text has been edited as shown below.**

***“Participants will also be classified based on whether they achieve the minimal clinically important difference (MCID) on relevant outcome measures….Differences in the proportion of participants in each group achieving these clinical importance thresholds will be presented using risk ratios and the number needed to treat (with associated 95% confidence intervals).~~, with significance determined using chi-square (χ²) analyses.”~~* (Lines 378-380)**

8. The authors did not mention the statistical tool for mediation analysis. This must be included in the manuscript.

**Response: Thank-you for this suggestion. It has been incorporated.**

**Action: The following text has been expanded to indicate the tool to be used for mediation analysis.**

***“Mediation analyses will be conducted to explore the possible mechanisms through which individualised physiotherapy exerts its effects. Linear regression models will be fitted using IBM SPSS to determine direct versus indirect (via a mediator variable) effects contributing to the total treatment effect”.* (Lines 395-397)**

9. The authors did not include the statistical tool for assessing effect modification. Thy only mentioned the effect modifiers. This must be included in the manuscript.

**Response: Thank-you for this suggestion. It has been addressed as suggested below.**

**Action: The manuscript has been modified as indicated below:**

***“Analyses of treatment effect modifiers will be conducted to identify baseline participant characteristics associated with greater or lesser benefit from individualised physiotherapy compared to usual care. Using linear mixed effects models (IBM SPSS), effect modification will be explored using a three-way interaction term (predictor x group x time)”.* (Lines 386-387)**

**Reviewer #2:**

1.In exclusion criteria they mentioned minimal physical activity limitation will be excluded. From my point there is no need to mention it rather than mention the minimal physical activity limitation criteria like can sit more than 1 hour without pain etc.

**Response: Thank-you for this suggestion. This change has been incorporated in Table 1.**

**Action: In Table 1, exclusion criterion 7, the text has been changed to:**

***“*~~Minimal activity limitation, evidenced by a baseline~~ ~~a~~*Ability to walk, sit, and stand for one hour or more and no sleep disturbance at night, as we wish to exclude people with low severity”***

2. Usual Care: Description of usual care should be included

**Response: As the nature of usual care modalities will be fully at the discretion of the treating physiotherapists at JSS Hospital, it is not possible to describe this treatment ahead of the trial being conducted. We will report on the specific treatment modalities applied by the usual care physiotherapists when the results of the trial are published.**

**Action: The manuscript has been modified as indicated below:**

***“Usual care group: Treatment provided to participants in this group will be at the discretion of their treating physiotherapist in accordance with their standard practice. These physiotherapists will not receive training in the STOPS approach. Specific treatments administered will be documented for each participant and reported with trial results”.* (Line 215)**

3. Who will be the treatment provider: You mentioned only 2 PT trained about STOP approach. Is it possible to serve only by them.

**Response: Given the extensive training requirements for the two registered physiotherapists in India who will deliver STOPS physiotherapy (including travelling to Australia for training), only two treatment providers will deliver treatment to the 77 participants receiving STOPS individualised physiotherapy. This is acknowledged in the discussion as a limitation regarding generalisability of findings to other settings and to other therapists, but is deemed suitable for an initial evaluation.**

***“This restricts the generalisability of findings to a limited participant population, therapist pool, and treatment setting. However, the trial provides an opportunity to evaluate the outcomes and feasibility of implementation before dedicating additional resources to wider implementation across multiple centres in India”.* (Lines 490-493)**
